# Synchrotron beamline setup enabling quasi-simultaneous PXRD and XANES measurements: case study of Fischer–Tropsch catalyst reduction at 60 bar

**DOI:** 10.1107/S1600577526003656

**Published:** 2026-06-02

**Authors:** Lipeng Yao, G. Leendert Bezemer, Irene. M. N. Groot, Oleg Konovalov, Maciej Jankowski

**Affiliations:** a Leiden Institute of Chemistry Leiden University Einsteinweg 55 2333 CCLeiden The Netherlands; b ESRF – The European Synchrotron, 71 Avenue des Martyrs, CS 40220, 38043Grenoble Cedex 9, France; c Shell Global Solutions International BV, Energy Transition Campus Amsterdam, Grasweg 31, 1031 HWAmsterdam, The Netherlands; Bhabha Atomic Research Centre, India

**Keywords:** *in situ* PXRD, *in situ* XANES, cobalt reduction, catalyst structure, beamline setup

## Abstract

Powder X-ray diffraction (PXRD) and X-ray absorption near-edge structure (XANES) spectroscopy are complementary techniques for probing cobalt-based (Co-based) Fischer–Tropsch synthesis (FTS) catalyst structures. PXRD reveals crystalline composition, while XANES provides information on coordination geometry and oxidation state. We developed a fast, automated measurement method, based on rapid selection of X-ray beam energy and a dedicated sample environment at beamline ID10 (ESRF), to combine *in situ* PXRD and XANES in a single experiment. This approach enables simultaneous monitoring of structural and electronic changes in Co-based FTS catalysts under *in situ* conditions up to 60 bar, offering a comprehensive view of catalyst dynamics.

## Abstract 2

This paper presents a beamline setup that allows the combination of *in situ* powder X-ray diffraction (PXRD) and X-ray absorption near-edge structure (XANES) measurements within a single experimental cycle to investigate cobalt catalyst structures and properties under real conditions.

## 1. Introduction

Synchrotron radiation, due to its high brilliance, tunable energy and excellent time resolution, enables powerful experimental methods for studying catalysts (Meirer & Weckhuysen, 2018 ▸). Advanced techniques, such as powder X-ray diffraction (PXRD) and X-ray absorption spectroscopy (XAS), including X-ray absorption near-edge structure (XANES) and extended X-ray absorption fine structure (EXAFS), are used to probe both structural and electronic changes in real time. This capability enables *in situ* and *operando*, *i.e.* under realistic reaction conditions, studies of dynamic processes, such as phase transitions, oxidation–reduction cycles, and metal–support interactions (Roldán Cuenya & Bañares, 2024 ▸; Chen *et al.*, 2024 ▸; van Ravenhorst *et al.*, 2021 ▸; Loewert *et al.*, 2020 ▸; Moya-Cancino *et al.*, 2019 ▸), which are often not feasible with commonly used laboratory methods. In industry, Fischer–Tropsch synthesis (FTS) is considered a green, environmentally friendly, and sustainable process for producing high-value chemical products such as waxes and liquid fuels from natural gas, waste and biomass (Rommens & Saeys, 2023 ▸). Among the various catalysts used, cobalt-based (Co-based) catalysts are particularly important due to their high activity, excellent selectivity toward long-chain hydro­carbons, and superior stability under typical FTS operating conditions (Davis, 2007 ▸). Synchrotron-based measurements provide critical insights into the relationships between catalyst structure, oxidation state and performance, making them an important tool for studying Co-based FTS catalysts and guiding rational catalyst design.

PXRD is one of the most widely used techniques for determining the crystallographic structure of heterogeneous catalysts, which are often in the form of nanometre-sized metallic nanoparticles (NPs) dispersed on various supports, typically oxide microcrystal surfaces (Holder & Schaak, 2019 ▸). It provides essential information about the structure, phase, composition, shape, size, and crystallinity of both active phase and support material (Kaduk *et al.*, 2021 ▸). Advanced data analysis, including Rietveld refinement (Rietveld, 1969 ▸), enables the determination of multiple structural parameters describing the investigated catalyst. The high brilliance of modern synchrotron sources, *e.g.* the ESRF–EBS upgrade (Raimondi, 2016 ▸), in connection with large-area 2D detectors (François, 2023 ▸) allows for the collection of PXRD patterns with very high rates and broad reciprocal space range (He, 2003 ▸), and enables real-time monitoring of the catalyst crystallographic structure as a function of the changeable reaction conditions. There are multiple examples of the application of PXRD to study Co-based catalysts during FTS reactions (Bo *et al.*, 2025 ▸). For instance, Tsakoumis *et al.* (2016 ▸) employed it *in situ* to elucidate the phase evolution during the reduction of an Re-promoted Co/Al_2_O_3_ catalyst, and the investigation of alumina-supported cobalt catalyst evolution under realistic conditions of FTS (Karaca *et al.*, 2010 ▸).

Another widely used method at synchrotrons for studying Co catalysts is XAS, which complements PXRD measurements by providing information about the chemical state of the active phase and promoters. It is a frequently used technique to elucidate the relationship between structure and catalytic performance in heterogeneous catalysis (Beaumont, 2020 ▸). *In situ* XAS enables the monitoring of local structural changes during reactions, with characteristic timescales ranging from minutes to tens of milliseconds (Frenkel, 2012 ▸). An example of an XAS application in FTS is a study by Meng *et al.* (2021 ▸) that investigated the structural dynamics of Ru species during high-temperature reduction and FTS reactions on Ru_1_Co_*n*_ single-atom alloy catalysts. Synchrotron-based *in situ* XAS was employed to examine the structural evolution of both cobalt- and lanthanide-containing phases during activation and under CO hydrogenation conditions (Ribeiro *et al.*, 2020 ▸). Another example was reported by Liu *et al.* (2019 ▸), who investigated the formation of cobalt carbide (Co_2_C) under *operando* conditions during FTS using *in situ* XAS.

Performing PXRD and XANES measurements independently may miss the direct correlations between structural and electronic changes under real reaction conditions. PXRD is insensitive to amorphous or poorly crystalline species, whereas XANES captures oxidation-state and coordination changes but does not provide information about phase composition. Separated measurements can lead to unclear interpretation in systems where multiple phases coexist, where short-lived intermediates form, or where sample conditions may change between experiments (*e.g.* exposure to air, temperature drift or beam effects). These limitations lead to unaccurate assignment of electronic states to specific crystalline phases and cannot give critical mechanistic insights. Combining *in situ* PXRD and XANES can directly monitor transient species, follow the evolution of structural and electronic states in real time, and establish more accurate structure–activity relationships. The combination of PXRD and XANES in a single experiment is also well suited to providing a more comprehensive overview of the structure and kinetics in heterogeneous catalysis or other materials (Sekizawa *et al.*, 2017 ▸; Frenkel *et al.*, 2011 ▸; Ehrlich *et al.*, 2011 ▸; Hamdalla *et al.*, 2023 ▸; Black *et al.*, 2024 ▸).

We developed a fast beam-alignment switching method to perform *in situ* PXRD and XANES within a single experimental cycle at the ID10-SURF beamline at the ESRF. This setup enables rapid, sequential PXRD and XANES scanning, allowing simultaneous monitoring of both structural and electronic changes in catalysts. This approach provides detailed insights into catalyst structure, oxidation state, and functional properties by capturing dynamic transformations. The combined techniques overcome the limitations of performing PXRD and XANES separately, enabling accurate observation of transient states and fast processes, making it a powerful tool for studying catalysts under *in situ* conditions.

To enable probing the Co-based model catalyst under conditions close to those used in industry (Dry, 1982 ▸), we have developed a dedicated sample environment capable of controlling gas composition, flow, and humidity at high pressures (up to 60 bar), as well as sample temperature from room temperature to 600°C. The merging of synchrotron-based experimental methods and such a dedicated sample environment, which enables reaching conditions that mimic those used in industry, provides a unique opportunity for *in situ* investigations of cobalt catalysts during FTS.

## 2. Technical details

### 2.1. Beamline setup

**Figure 1:**
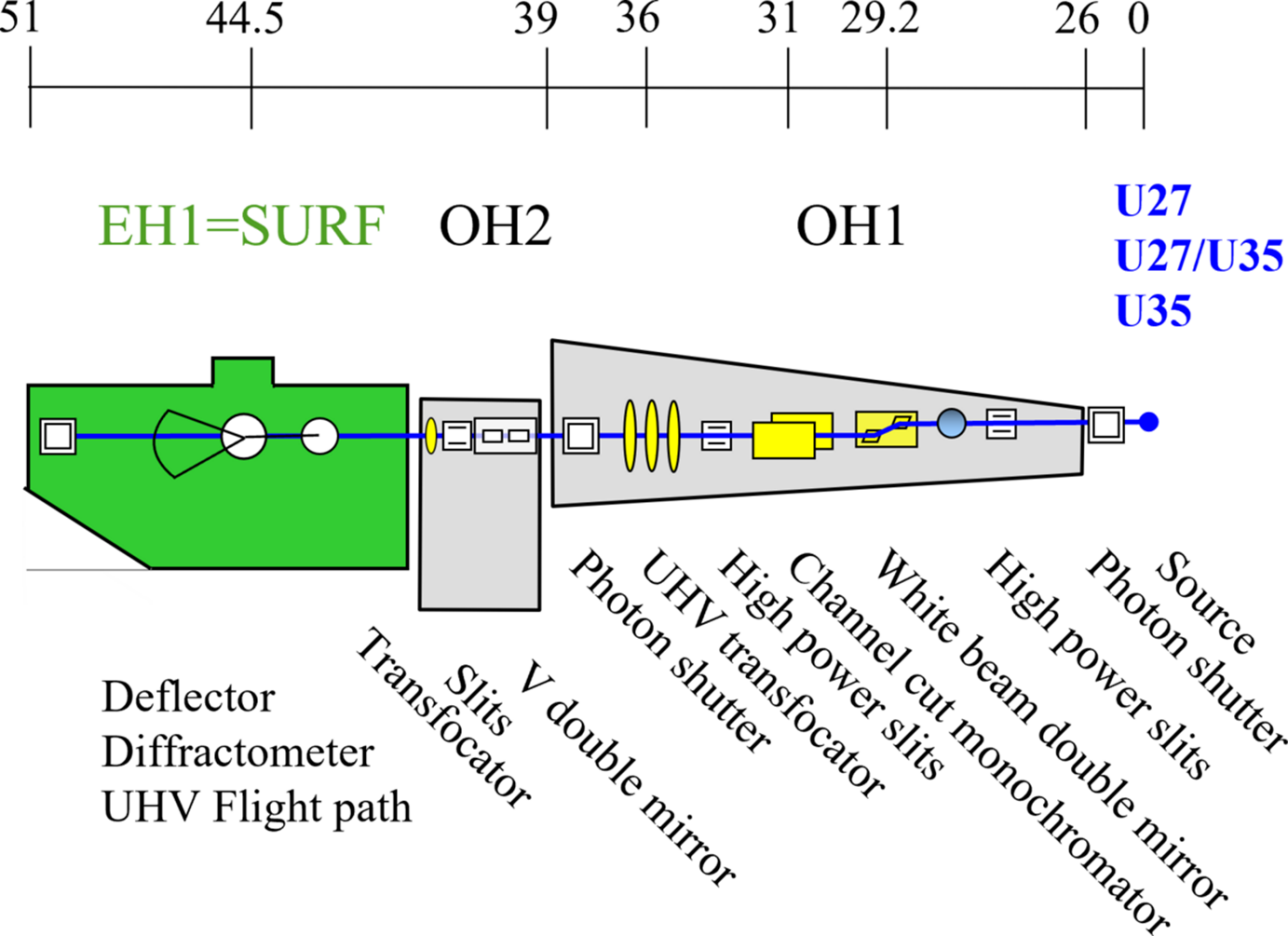
Schematic view of the ID10-SURF beamline optical layout. The insertion devices are two undulators, U27 and U35, and two U27/U35 mounted in the revolver. The beamline is composed of optical hutch 1 and 2 (OH1, OH2), and experimental hutch 1 (EH1), dedicated to investigating surfaces and interfaces (U27: undulator with a 27 mm magnetic period; U35: undulator with a 35 mm magnetic period).

The ID10-SURF beamline is dedicated to high-resolution X-ray scattering and surface diffraction at liquid and solid interfaces, integrating multiple techniques within a single instrument (Jankowski *et al.*, 2023 ▸; Narayanan & Konovalov, 2020 ▸). The beamline optics layout is shown in Fig. 1 ▸. The beamline is equipped with a double-crystal deflector coupled to a 6+2 diffractometer, allowing for high-resolution X-ray scattering at grazing incidence in various horizontal and vertical geometries (Konovalov *et al.*, 2024 ▸). This setup allows for multiple scattering geometries, the use of different sample environments, and the investigation of samples ranging from powders (presented in this work) to single-crystal surfaces (Templeman *et al.*, 2019 ▸), thin films (Pithan *et al.*, 2023 ▸), liquid surfaces (Maiti *et al.*, 2023 ▸) and liquid metal surfaces (Jankowski *et al.*, 2021 ▸).

**Figure 2:**
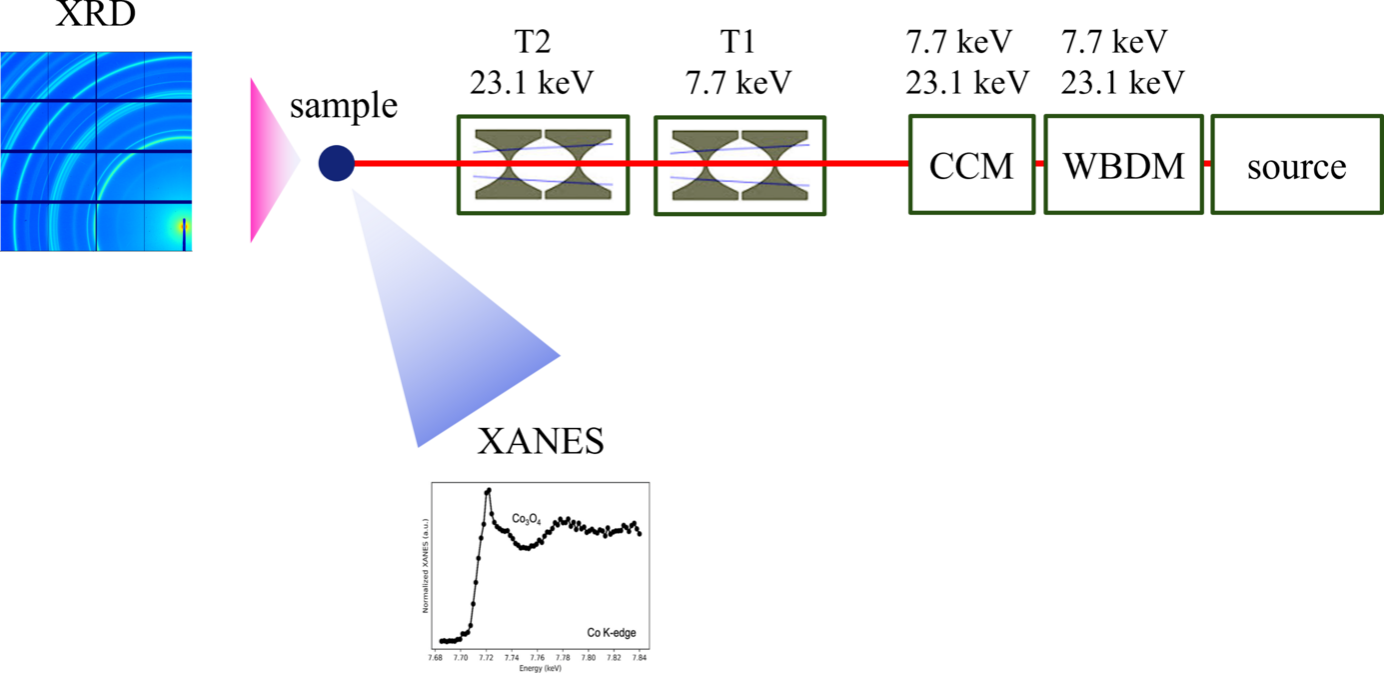
Quasi-simultaneous XRD and XANES measurements layout using two transfocators. It includes a white-beam double mirror (WBDM) for higher harmonic rejection and a channel-cut Si monochromator (CCM) for energy selection. Two transfocators, T1 (OH1 transfocator) and T2 (OH2 transfocator), are used to dynamically focus and shape the beam at different energies. The precise positions of the two transfocators along the beamline are illustrated in Fig. 1 ▸. The XRD signal is collected by a detector positioned in front of the sample, while the XANES signal is collected by the fluorescence detector, which is placed behind the sample at a 45° angle relative to the incident X-ray beam.

The beamline beam energy can be tuned between 7 and 30 keV by using three X-ray undulators (U27, U27/U35 – a revolver unit carrying both U27 and U35 undulators, and U35) (Willmott, 2019 ▸) and a Si(111) channel-cut monochromator as shown in Fig. 2 ▸. In our case, we tuned the undulators to maximize the flux of the first undulator harmonic at 7.708 keV, corresponding to the Co absorption edge. The fifth and higher harmonics of the X-ray beam are rejected by setting the palladium-coated double mirrors at an appropriate grazing angle (typically 2.5 mrad). This mirror is placed in the optical hutch right after the primary slits and before the monochromator. The X-ray beam composed with the first and third harmonics of the undulator is monochromated further by the channel-cut monochromator. The monochromator angle is optimized for scattering of the third harmonic on Si(333) crystallographic planes while the first harmonics, following Bragg’s law, are naturally monochromated by scattering on the Si(111) crystallographic planes as at the Bragg angle these energies and scattering planes are equal. As a result, the X-ray beam after the monochromator is bichromatic with two wavelengths corresponding to 7.7 keV (the first harmonics) and 23.1 keV = 3 × 7.7 keV (third harmonics). The beam was focused using two transfocators filled with a Be compound refractive lens (CRL). The first transfocator (T1, OH1 transfocator), located between the X-ray source and the sample at 1:11 demagnification position, used a set of four beryllium lenses with a curvature radius of 200 µm to focus the 7.7 keV beam on the sample for XANES measurements near the cobalt edge. The second transfocator (T2, OH2 transfocator), located at 1:4 demagnification position, used 32 beryllium CRLs with a radius of 300 µm to focus the 23.1 keV beam on the sample. The latter was used for PXRD measurements. The X-ray beam was focused by inserting one of two sets of CRLs into the X-ray beam path corresponding to the best focus at given energy: T1 set at 7.7 keV used for XANES, and T2 set at 23.1 keV used for PXRD. Switching between the two focusing configurations takes approximately 1 s, allowing rapid changes in beam energy without the need to retune the undulators, monochromator, and all the rest of the beamline setup. Using such configurations, we are able to follow any system evolution on the minute timescale using switching between PXRD and XANES configurations. A slit with an aperture of 0.1 mm × 0.1 mm (V × H) located 200 mm before the sample allows only the focused beam to be selected. When 7.7 keV is focused, the major part of the non-focused high energy, 23.1 keV, is blocked by this slit. The contribution of the remaining part of the high energy to the XANES signal is negligible. When the high energy, 23.1 keV, is focused, the low-energy part of the beam is completely defocused by 32 CRLs and the remaining part of this energy passing through the slits is fully attenuated by the sample and so does not contribute to the PXRD pattern. The beam size at the sample’s position was 51 µm × 44 µm and 31 µm × 13 µm at 7.7 and 23.1 keV, respectively. The corresponding flux is ∼1.9 × 10^12^ photon s^−1^ (7.7 keV) and 2.1 × 10^10^ photon s^−1^ (23.1 keV), both at a synchrotron storage ring current of 100 mA. PXRD and XANES measurements were performed sequentially to enable time-resolved correlation between electronic and structural changes. The XANES measurements were performed by scanning the beam energy in the range 7.65–7.85 keV using the monochromator. After each XANES scan, the monochromator returned rapidly to 23.1 keV for PXRD acquisition. Each XANES scan was completed within approximately 5 min, and the corresponding PXRD pattern was recorded in ∼20 s. The rapid alternation between XANES and PXRD measurements provided high temporal resolution in tracking the dynamics of structural and electronic changes. It is worth mentioning that, in principle, extending our method to EXAFS measurements would be possible, but would require modifications to our undulator mechanics and optics to allow continuous adjustment of the undulator gap to maintain a sufficient photon flux for scans with extended energy ranges and to provide reasonable scan times. Currently, such scans are offered by dedicated spectroscopy beamlines more suitable for *operando* EXAFS measurements.

### 2.2. Cell design

**Figure 3:**
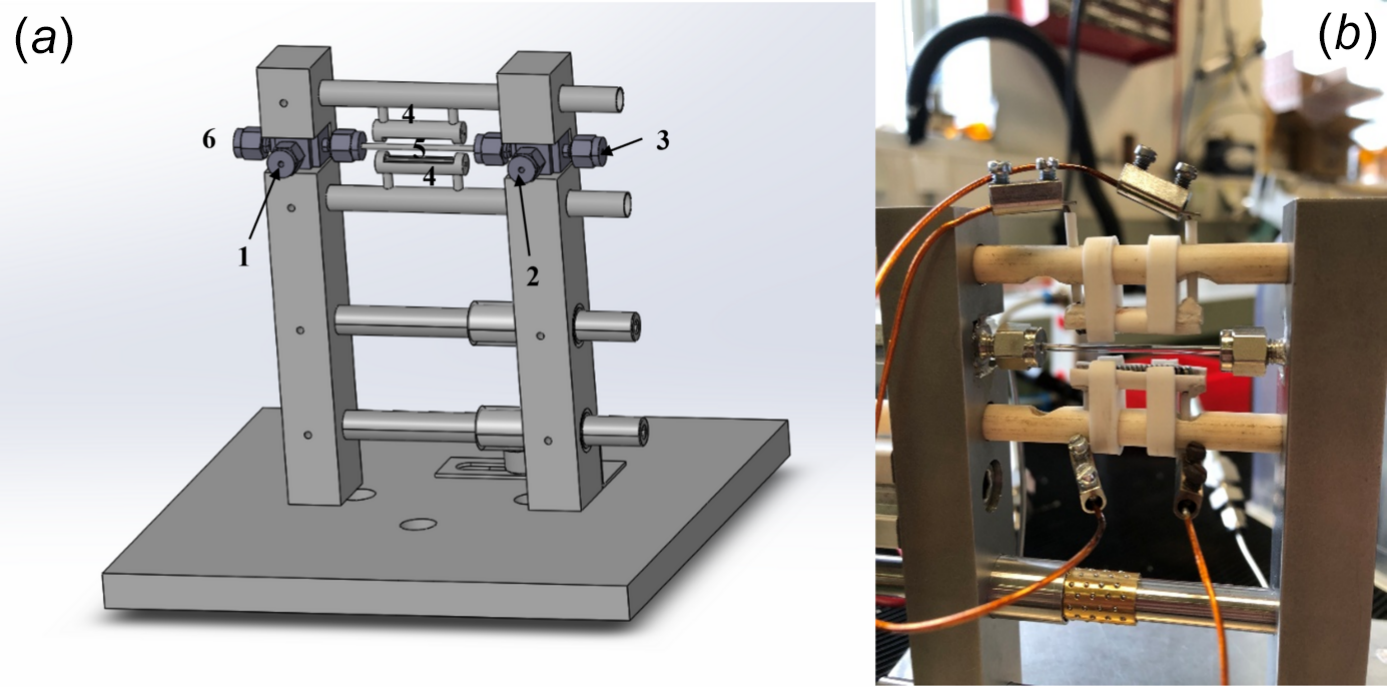
(*a*) Three-dimensional (3D) schematic model of the reaction cell showing key components: (1) gas inlet; (2) gas outlet; (3) thermocouple, positioned to monitor the temperature accurately; (4) resistive coils, heating to desired reaction temperatures; (5) capillary, allowing access for *in situ* measurements in continuous gas flow conditions; and (6) metal gasket face seal fittings, ensuring a hermetic and high-pressure-resistant seal. (*b*) Photograph of the assembled reaction cell, illustrating the physical arrangement, corresponding directly to the 3D model in (*a*).

The sample cell (Fig. 3 ▸) was designed in collaboration with the ESRF sample environment group. The cell allows for working with the catalyst placed inside a thin-walled capillary that permits controlled gas flow through the sample. The experiments are performed in continuous-flow mode, in which a gas mixture flows continuously through the capillary, and the capillary pressure is set by one of the pressure regulators placed after the cell. The pressure regulators allow experiments to be conducted in two pressure regimes: 1–13 bar or 13–60 bar. The capillary is held between two metal supports of which one side is adjustable, enabling precise alignment and stable mounting during experiments. Fig. 3 ▸(*a*) shows a 3D model of the cell and Fig. 3 ▸(*b*) shows a photograph of the as-built cell. The cell was tested at a maximum pressure of 200 bar for safety testing, a continuous operation pressure of 60 bar, and sample temperatures up to 600°C. Radiative heating was achieved using resistive coils positioned above and below the capillary, ensuring a stable and uniform temperature, similar to the twin-coil design reported by Chupas *et al.* (2008 ▸). For accurate temperature measurement, a K-type chromel–alumel thermocouple with a diameter of 0.25 mm was inserted directly into the catalyst bed, enabling precise monitoring of the catalyst’s temperature. The sample alignment is performed in the X-ray beam’s transmission mode, allowing accurate localization of the thermocouple position. Therefore, the sample spot probed by the X-ray beam during the experiment can be positioned very close to the thermocouple, typically within 1–2 mm, ensuring that the difference between the readout temperature and the temperature at the probed spot is negligible. A capillary [∼1.6 mm outer diameter (OD) and 1.2 mm inner diameter (ID)] from Hilgenberg (quartz for low pressure) or Crytur (∼1.6 mm OD and 1.2 mm ID sapphire for high pressure) was mounted in a T-type connection using graphite capillary ferrules, with the thermocouple sealed into the powder sample on one side using another graphite ferrule. During laboratory preparation experiments, both quartz and sapphire capillaries were tested over a range of pressures. The quartz capillary demonstrated pressure resistance up to 30 bar with gas flow and heating at 400°C for two days without failure. The sapphire capillary was exposed to the same test protocol, but with a maintained pressure of 200 bar. These requirements were set by safety protocols, and special considerations are needed for the *operando* experiments. During these experiments, measurement parameters such as acquisition time per scan, beam energy, energy scan step resolution, temperature ramp, and gas pressure must be aligned with capillary parameters. This alignment allows monitoring of the catalyst’s phase-change kinetics within a reasonable time frame and ensures that the recorded spectra are of adequate quality. In our measurement, the XANES signal was attenuated by a factor of 6.8 × 10^−2^ due to the capillary wall thickness, but thanks to the high photon flux of the ID10-SURF beamline the measurements remained feasible. For the PXRD measurement at 23.1 keV, the attenuation is significantly lower, enabling the powder diffraction spectra to be recorded in seconds. Heating power was controlled by a proportional-integral-derivative (PID) controller (Nanodac, Eurotherm).

### 2.3. Gas system

**Figure 4:**
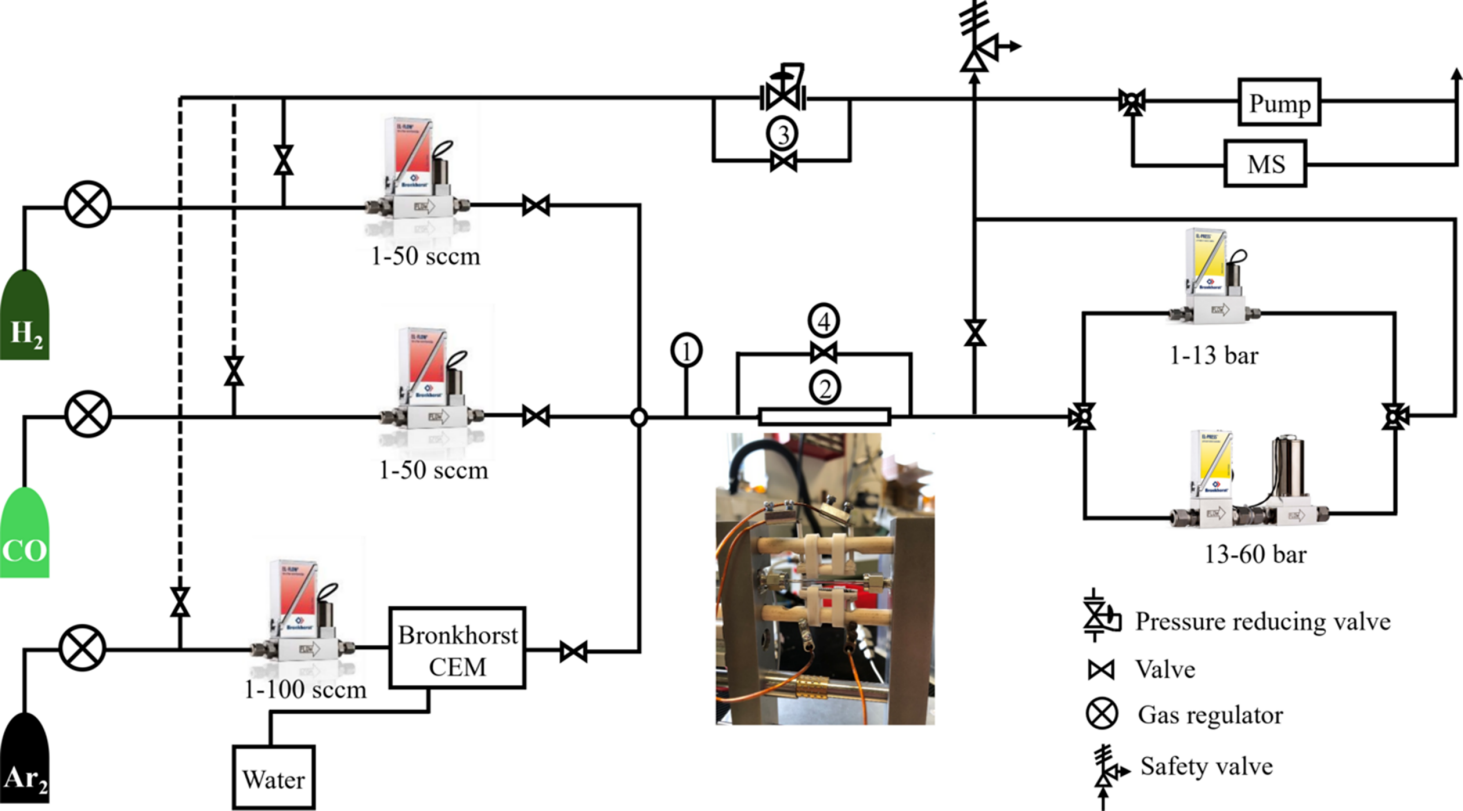
Detailed schematic of the gas panel setup. The system includes: (1) pressure manometer for monitoring the gas pressure; (2) reaction cell; (3) bypass-1; and (4) bypass-2. Gas flow is controlled using mass flow controllers: MFC-1 for H_2_, MFC-2 for CO, and MFC-3 for Ar. Pressure regulation is achieved using two stages: low-pressure control (1–13 bar) via pressure regulator-1 and high-pressure control (13–60 bar) via pressure regulator-2. This configuration allows precise control of gas composition, flow rates, and operating pressure during experiments.

The gas panel setup (Fig. 4 ▸) was designed in collaboration with the Swagelok company. Gas flow and composition were precisely controlled using three mass flow controllers (MFCs) from Bronkhorst, The Netherlands, connected to individual gas cylinders, with downstream check valves installed to prevent backflow. The H_2_ and CO mass flow controllers have a maximum flow rate of 50 standard cubic centimetres per minute (sccm), while the N_2_ mass flow controller can reach up to 100 sccm. Reactor pressure can be regulated separately by two pressure regulators (1–13 bar, low pressure; 13–60 bar, high pressure), each managed by a process controller. To control gas humidity, the system allows injection of humid inert gas via a controlled evaporator mixer (CEM) (Bronk­horst, The Netherlands) connected to a pressurized water tank. The water flow range is from 0 to 2 g h^−1^. Using this approach, water vapor can be injected into the main gas stream even at 60 bar. All inlet and outlet lines can be heated to 200°C using insulated lines to prevent water vapor condensation. An empty collection tube downstream of the cell captures water originating from the water vapor passing through the sample cell, and this is produced during the reaction. A bypass-1 valve is located next to the safety valve to quickly release pressure in case of excessive system pressure. The gas composition in the low-pressure section is analyzed by mass spectrometry (LPM, T100 Gas Analyzer). To protect the mass spectrometer and ensure overall system safety, a safety valve is installed downstream of the pressure regulator. This valve prevents over-pressure conditions in the low-pressure section and is set to relieve at 2 bar. It is calibrated to open automatically when the internal pressure reaches 2 bar, preventing the pressure from rising to levels that pose a safety risk to operators. Another bypass-2 valve is installed in parallel with the reactor, next to the sample cell. This bypass valve can prevent samples from being drawn into the gas pipe when the system is pumped. Analytical devices, such as mass spectrometers, can be used to monitor the composition of the flowing gas and quantify catalyst performance. All system parameters are monitored and recorded using the ESRF-BLISS control system (Guijarro *et al.*, 2023 ▸).

## 3. *In situ* techniques

### 3.1. *In situ* PXRD

PXRD scattering data were collected using an Eiger CdTe 4M 2D detector, placed at the position shown in Fig. 2 ▸. The 2D diffraction images were azimuthally integrated using *pyFAI* (Ashiotis *et al.*, 2015 ▸) and subsequently analyzed with *GSAS-II* (Toby & Von Dreele, 2013 ▸). The incident X-ray wavelength was set to λ = 0.536 Å. Before data collection, the detector position was calibrated using an LaB_6_ standard (NIST SRM 660) and *pyFAI* calibration module. For each measurement, the 2D detector image was recorded for 20 s and integrated to obtain diffraction data. The resulting diffractograms of the measured samples were analyzed using the Rietveld method, with TiO_2_ anatase, rutile, CoO, Co_3_O_4_, Co-f.c.c. and Co-HPC phases included in the fitting.

### 3.2. *In situ* XANES

XANES spectra were collected over the energy range 7650–7850 eV with 2 eV step increments and an integration time of 3 s per point (2 eV step), resulting in a total acquisition time of approximately 5 min per spectrum. Measurements were performed in fluorescence mode using a silicon drift detector (SDD, Vortex-90EX) with a 90 mm^2^ active area, positioned 20 mm from the sample at a 45° angle relative to the incident X-ray beam, as shown in Fig. 2 ▸. All spectra were processed using custom Python scripts that included background subtraction and normalization based on the pre-edge and post-edge regions to facilitate an accurate comparison of edge features. The measurement parameters were chosen to include multiple factors required for the *operando* measurements, balancing the length of the scan and the quality of the recorded spectra. These are the number of points per scan, the energy step, the temperature ramp rate, the reaction kinetics speed, the amount of measured material, the X-ray beam photon flux and its transmission through the sample capillary walls, gas pressure, and safety protocols. Thus, in this case, to balance all these factors, we chose step 2 eV, which allowed us to record XANES spectra within a 5 min window. This exceeds the theoretical resolution of the beamline monochromator (0.6 eV at 7.7 keV), but *operando* measurements often require careful testing and compromises, depending on the system under investigation.

### 3.3. Sample preparations and measurements

To evaluate the performance of the experimental setup, a sample of 10 wt% Co supported on TiO_2_-P25, promoted with 0.2 wt% rhenium (Re), was tested under N_2_/H_2_ reduction conditions at 60 bar. The catalyst was first gently crushed in a quartz mortar, and was sieved to obtain particles within the 100–120 mesh size range. A sample of around 10 mg was then loaded into a sapphire capillary, with both ends sealed with quartz wool to prevent sample displacement caused by gas flow. Finally, the capillary containing the sample was carefully mounted into the reactor cell.

Before starting the experiment, a gas leak test was performed using dedicated leak detectors. Then, the sample was dried under a flow of N_2_ (50 sccm) at 120°C for 30 min, followed by the injection of H_2_ into the gas stream. The reduction was carried out at 60 bar under an N_2_/H_2_ mixture (25/25 sccm) with a heating rate of 2°C min^−1^ from 120°C to 400°C. Throughout the entire reduction process, PXRD and XANES data were continuously recorded.

## 4. Results

**Figure 5:**
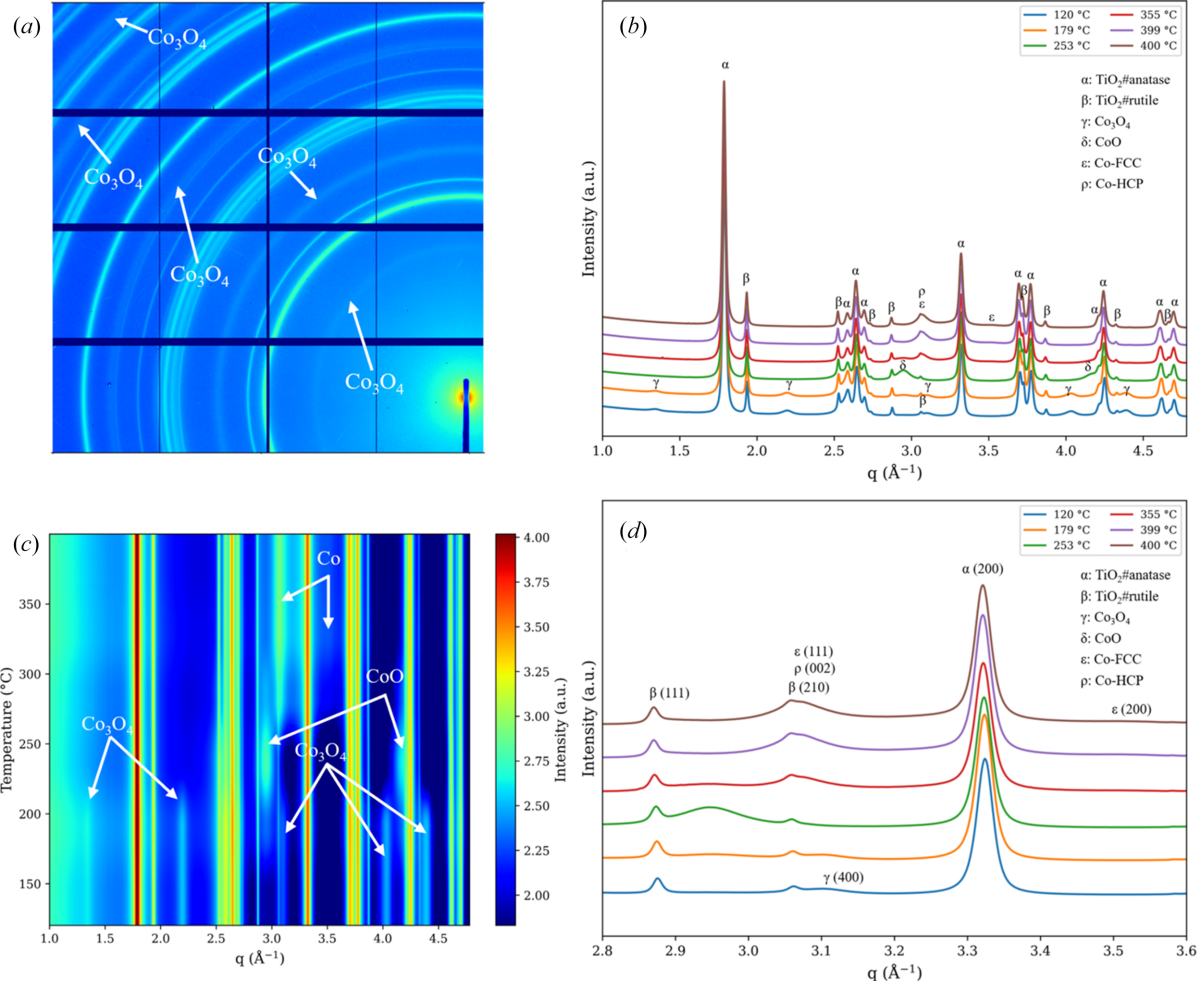
(*a*) Fresh sample 2D PXRD detector image. The white arrows mark the rings originating from the Co_3_O_4_ phase in the fresh, oxidized catalyst. (*b*) *In situ* PXRD 1D profiles plotted as a function of temperature; in the PXRD pattern at 120°C is the first PXRD scan after adding H_2_. (*c*) Integrated temperature-resolved PXRD patterns during the reduction process in N_2_/H_2_ at 60 bar. (*d*) Zoom PXRD patterns of Fig. 5 ▸(*b*) (2.8–3.6 Å^−1^ region).

Fig. 5 ▸(*a*) shows an example of a PXRD 2D pattern recorded by the detector for the fresh catalyst sample at 120°C. The broader rings, marked by the arrows on the 2D image, originate from Co_3_O_4_ nanoparticles, whereas the rest of the rings belong to the TiO_2_ support, including both anatase TiO_2_ and rutile TiO_2_. The integrated cobalt species diffraction patterns [as shown in Fig. 5 ▸(*b*)] show the PXRD patterns collected during the reduction process. The detailed phase reflections are presented in Table S1 of the supporting information. In the fresh sample, diffraction peaks corresponding to Co_3_O_4_ are observed at *q* = 1.34 Å^−1^, 2.20 Å^−1^, 3.10 Å^−1^, 4.03 Å^−1^ and 4.39 Å^−1^, which is a common state of FTS catalysts before the reduction (Prabaharan *et al.*, 2017 ▸). As the temperature increases to 200°C, the appearance of new peaks at 2.94 Å^−1^ and 4.16 Å^−1^ indicates the formation of CoO. This phase transformation is well known and has been reported as equation (1). Subsequently, the CoO is reduced to metallic cobalt as equation (2) (Pöyhtäri *et al.*, 2025 ▸),
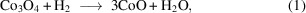

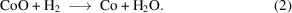
The dynamics of the process can be easily tracked; see Fig. 5 ▸(*c*), which shows the *operando* PXRD spectra plotted as a function of temperature in the 120°C to 400°C range. As shown in Fig. 5 ▸(*d*), both face-centered cubic (f.c.c.) and hexagonal close-packed (h.c.p.) cobalt phases are present, which are expected phases of the metallic Co catalyst (du Plessis *et al.*, 2016 ▸). The broad peak at *q* = 3.52 Å^−1^ is assigned to Co-f.c.c. (200), while an additional peak appears at *q* = 3.08 Å^−1^. Furthermore, an overlap between the Co-h.c.p. (002) and Co-f.c.c. (111) reflections is observed around *q* = 3.07 Å^−1^, along with a rutile-related peak as Fig. 5 ▸(*d*) shows.

**Figure 6:**
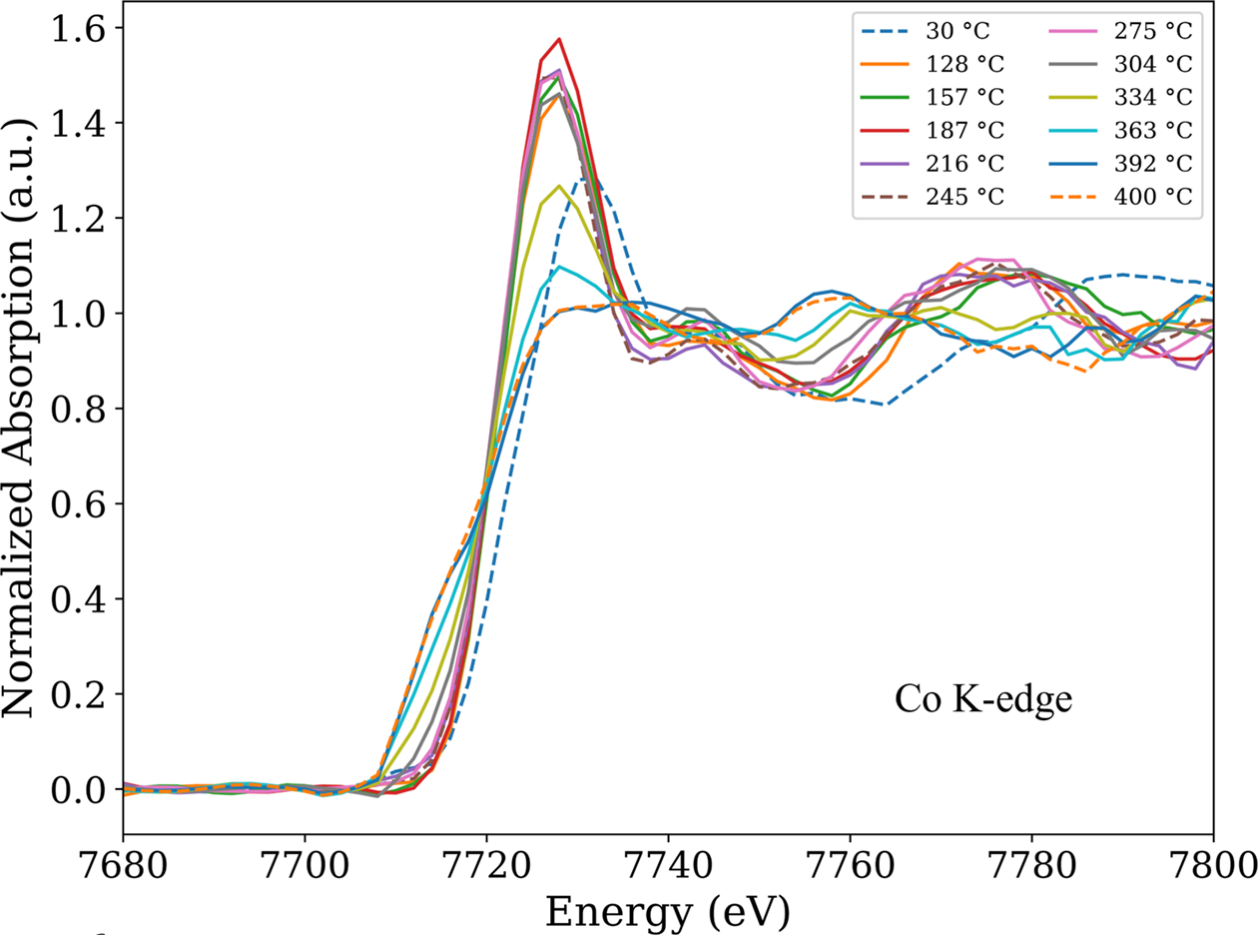
*In situ* XANES spectroscopy recorded at 30°C of the fresh sample for reference of Co_3_O_4_ and from 120°C to 400°C during the reduction process, showing the evolution of the cobalt oxidation state (Co_3_O_4_) to cobalt metal species over the temperature during the reduction process. The dashed lines are three different references for this experiment. The energy step in each XANES scan is 2 eV, and the total scan time per spectrum is 5 min.

Fig. 6 ▸ presents *in situ* Co *K*-edge XANES spectra collected under flowing gas at various temperatures. The initial spectrum, recorded at room temperature, is characteristic of Co_3_O_4_ (Jiang & Ellis, 1996 ▸), which contains both tetrahedrally coordinated Co^2+^ and octahedrally coordinated Co^3+^. This mixed-valence state results in the highest absorption-edge energy among the three phases, consistent with the predominance of Co^3+^. A pronounced white-line feature appears at ∼7725 eV, arising from the large number of unoccupied 3*d*- and 4*p*-derived states associated with Co^3+^ sites.

**Figure 7:**
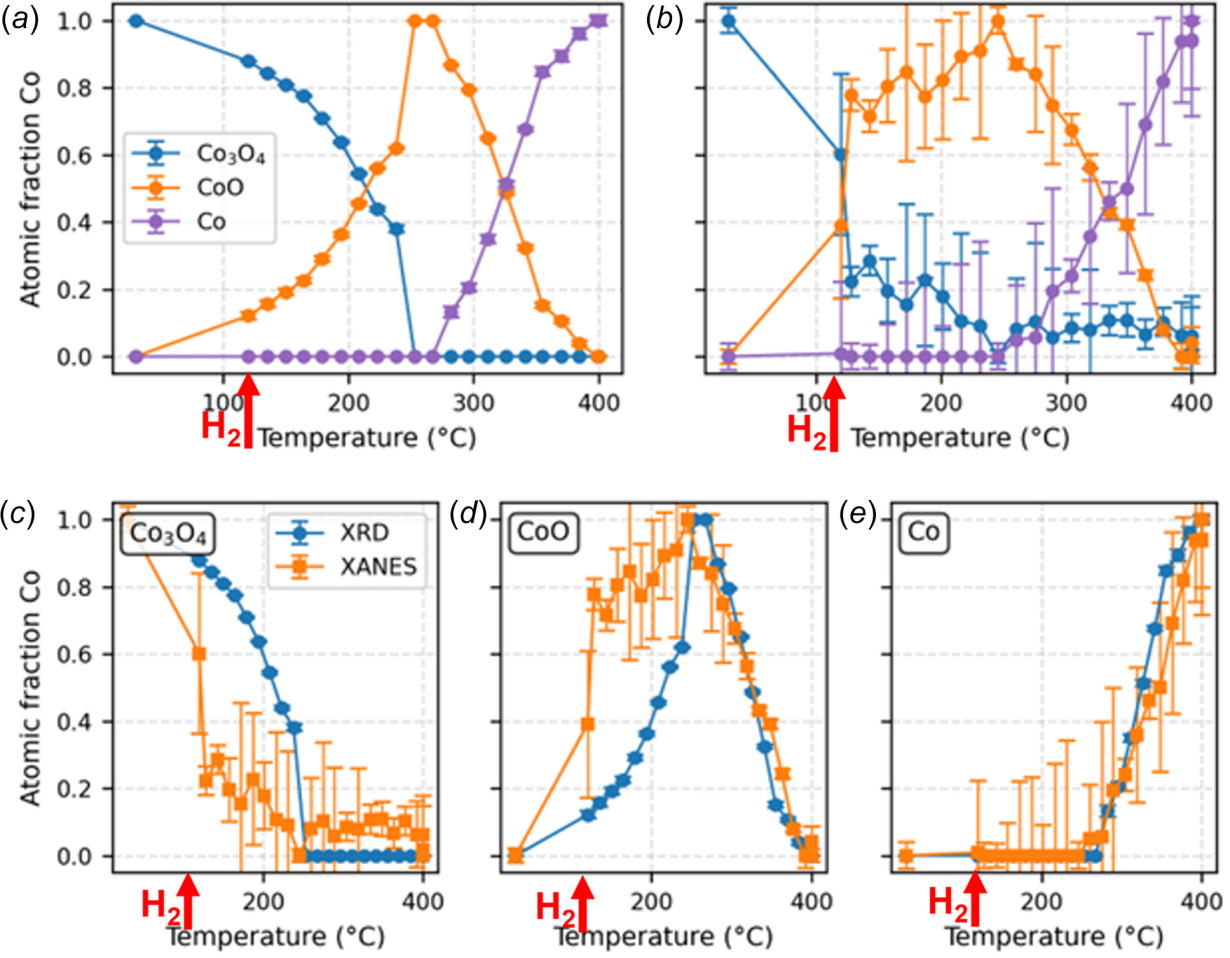
(*a*) Evolution of the atomic phase fraction content of Co species as a function of temperature, obtained from PXRD Rietveld refinement of the powder patterns presented in Fig. 5 ▸. (*b*) Evolution of the atomic phase fraction obtained from the XANES data using the linear combination fitting method. Panels (*c*), (*d*) and (*e*) compare the atomic fractions obtained from XRD and XANES measurements for Co_3_O_4_, CoO and metallic Co, respectively. Injection of H_2_ to the gas stream is marked by red arrows.

Upon heating, the spectra evolve and increasingly resemble that of CoO. The edge position shifts to lower energy, indicating the reduction of Co^3+^ to Co^2+^, accompanied by a substantial decrease in white-line intensity due to the lower number of unoccupied electronic states in Co^2+^. These spectral changes are characteristic of progressive cobalt reduction and are consistent with previous reports (Chen *et al.*, 2013 ▸). Quantitative Rietveld analysis was carried out, and the results are presented in Fig. 7 ▸ together with the XANES results. Results do indicate that already at 120°C, upon first introduction of hydrogen, CoO starts to form. This temperature is significantly lower than typically observed for supported cobalt catalysts and reflects the operating pressure of 60 bar as chosen in our experiments.

From 270°C onwards, further reduction leads to a second distinct spectral transition corresponding to the transformation of Co^2+^ into metallic Co. The absorption edge continues to shift toward lower energy, approaching that of Co metal, while the white-line feature becomes significantly weakened, reflecting the high occupancy of 3*d* states in metallic cobalt. The systematic evolution of these spectral features strongly supports the two-stage reduction pathway identified by the *in situ* PXRD measurements.

In Fig. 7 ▸(*a*), the evolution of the atomic phase fraction, derived from the Rietveld analysis of the PXRD spectra, is displayed. The reduction of Co_3_O_4_ follows a well known pathway, described above, in which the cubic spinel phase is reduced to the CoO rock-salt phase and, eventually, to metallic cobalt (80% Co-h.c.p.). Here, we combine the Co-h.c.p. and Co-f.c.c. phase contents to facilitate comparison with the XANES data. Metallic cobalt is observed from 270°C onwards. The low-temperature onset is attributed to the high pressure (60 bar) and the moderate heating rate of 2°C min^−1^. The PXRD provided information not only on the crystallographic phase content but also on particle sizes, lattice constants, and the support phase content, in which we detected the rutile and anatase phases of TiO_2_ (see Tables S2 to S9).

To determine the relative abundance of each cobalt species as a function of temperature from XANES measurements, linear combination fitting (LCF) of the XANES region was performed (see Table S10 for more details), and the goodness of each scan fitting is also shown in Fig. S1. As a reference for the spectra, we selected several spectra recorded during an *operando* run at temperatures at which only one phase was confirmed by PXRD. Using references extracted under the same sample and measurement conditions ensures that systematic offsets in edge energy, white-line shape, and beamline energy calibration are identical between the reference and sample spectra, minimizing a known source of error in linear combination fitting, see Fig. S2. The Co_3_O_4_ reference spectrum was taken from the fresh catalyst, whereas the CoO reference was obtained once Co_3_O_4_ was fully converted to CoO. A spectrum, recorded at 400°C after a prolonged period, served as the standard for metallic Co. However, the traditional approach of recording high-quality standard spectra from bulk material could potentially provide better LCF analysis; but caution is necessary because, under heterogeneous catalyst conditions, using bulk spectra might cause discrepancies (Dai *et al.*, 2017 ▸, Bazin & Rehr, 2003 ▸) due to different types of disorder, nanoscale features or dynamically changing structures that real working catalysts display. In our case, 0.2 wt% Re loading of the catalyst can already contribute to some of these discrepancies.

The LCF results, shown in Fig. 7 ▸(*b*), plot the cobalt oxidation state as a function of time and temperature. The Co_3_O_4_ → CoO transition starts at 120°C, just after injection of H_2_, and is completed near 270°C, followed by a gradual CoO → Co reduction occurring between 270°C and 400°C. The data enable evaluation of cobalt phase changes as a function of oxidation state. In Figs. 7 ▸(*c*)–7(*e*) we directly compare the evolution of atomic phase fractions derived from PXRD and XANES analysis. For the metallic phase, shown in Fig. 7 ▸(*e*), we observe good agreement in both the onset reduction temperature and the metallic fractions obtained. However, during the initial part of the reduction [Figs. 7 ▸(*c*)–7(*e*)], the discrepancy in CoO fractions is significant, even though the same temperatures were observed for the onset and completion of reduction. The difference in CoO fraction may be explained by XRD’s insensitivity to the formation of non-crystalline, amorphous oxide phases (Bates *et al.*, 2006 ▸), which could lead to an overestimation of the Co_3_O_4_ phase content; however, this would require more than 40% of the cobalt oxide phase to be amorphous. Based on our analysis and comparison of the %wt phase content of cobalt species with TiO_2_ (see Table S2), this explanation is unlikely. The discrepancy likely results from the choice of reference spectra and the high error in LCF analysis due to the quality of the recorded spectra, which is compromised by the measurement parameters, as noted in Section 3.2. On the other hand, such discrepancies are also reported for other techniques; for example, Plessis *et al.* (2013 ▸) reported phase-abundance discrepancies in a similar system, reaching 50% when comparing XRD and pair-distribution function (PDF) analyses.

Nevertheless, when combined with *in situ* PXRD measurements, structural information can be correlated to provide insight into the cobalt reduction process in H_2_. This approach enables monitoring and analysis of cobalt transformations at every stage of the process. By combining these two *in situ* synchrotron radiation characterization techniques, we can clearly capture structural and electronic changes in cobalt species during H_2_ reduction. Notably, certain variations can be deduced from PXRD patterns, *e.g.* crystallographic phase content, nanoparticle size, and lattice constant. The corresponding changes in phase content are also clearly resolved in the XANES spectra, highlighting the complementary sensitivity of these methods, especially where non-crystalline phases may be present.

XRD is sensitive to the crystallographic fraction of the catalyst and to long-range order, whereas XANES is an element-sensitive technique that probes electronic structure and is highly sensitive to the local, short-range coordination geometry around the target element atoms. This is very helpful for studying amorphous or doped phases and few-nm-sized nanoparticles, which have some degree of structural disorder, a high surface-to-bulk ratio, and much poorer crystallinity than their counterparts. Combining these two techniques in a single *operando* experiment can provide information on phase evolution as a function of the experimental conditions. In the literature, there are reports where a comparison of the results obtained from the two different methods often leads to quite significant discrepancies in the case of Co-based Fischer–Tropsch catalysts, *e.g.* cobalt carbide formation was detected earlier during FTS by XANES than by XRD (Rønning *et al.*, 2010 ▸); the CoO phase during the reduction to CoO could still be detected by XANES below 10% of phase content whereas XRD could not (Beale *et al.*, 2017 ▸); XANES detects a significantly thicker oxidized Co region than XRD because it captures non-crystalline and nanoscale CoO species that are invisible to diffraction, leading to an apparent smaller metallic Co fraction compared with XRD. Similar results also were observed by du Plessis *et al.* (2013 ▸), mentioned above, who used scattering methods like XRD and PDF to show that there is a significant discrepancy between the two methods in estimating phase content.

As further evidence for the complementary nature of PXRD and XANES measurements, we point to our observations at the end of the experiment where we performed a passivation treatment with diluted oxygen at room temperature to allow exposure to air without causing extensive cobalt oxidation. During the passivation treatment, as determined from XANES, the formation of a thin protective cobalt oxide layer on the metallic particles was shown (Wolf *et al.*, 2016 ▸) [see Fig. S3(B)]. The accompanying PXRD [see Fig. S3(A)] measurements did not pick up any formation of CoO. Apparently, the oxidic cobalt layer is too thin, and was not sufficiently ordered to yield the CoO signal, even though the total contribution was sizeable.

## 5. Summary and conclusion

The proposed approach demonstrates the potential of combining time-resolved PXRD and *in situ* XANES to study catalysts under real reaction conditions. Even though the ID10-SURF beamline concentrates on PXRD and X-ray scattering measurements, its flexible X-ray optics and diffractometer configuration enable auxiliary XANES measurements that complement the PXRD data, using a dedicated setup for high-pressure catalytic studies of powdered samples. This is achieved by minimizing interference with the beamline optical alignment, enabling fast switching between two measurement modes. With the proper design of the new sample cells, this method could be extended to other systems, *e.g.* solid-state and liquid surfaces and interfaces studied in catalysis or electrochemistry (Prajapati *et al.*, 2025 ▸; León & Mozo, 2018 ▸). This approach provides a deeper understanding of both the structural evolution and reaction kinetics of a catalyst during chemical processes, significantly enhancing the ability to investigate catalytic behavior during real chemical reactions.

## Supplementary Material

Tables S1 to S10 and Figs. S1 to S3. DOI: 10.1107/S1600577526003656/ye5081sup1.pdf

## Data Availability

The experimental data used in the present paper can be made available after the first author’s PhD thesis submission.
