# Supplementary material for: Synchrotron beamline setup enabling quasi-simultaneous PXRD and XANES measurements: case study of Fischer–Tropsch catalyst reduction at 60 bar
Source: J Synchrotron Radiat. 2026 Jun 2;33(Pt 4):968–77. doi: 10.1107/S1600577526003656 (PMC13344594; doi:10.1107/S1600577526003656)
Supplement: Supplementary file 1 [file s-33-00968-sup1.pdf]

JOURNAL OF SYNCHROTRON RADIATION

Volume 33 (2026)

Supporting information for article:

Synchrotron beamline setup enabling quasi-simultaneous PXRD and XANES measurements: case study of Fischer–Tropsch catalyst reduction at 60 bar

Lipeng Yao, G. Leendert Bezemer, Irene. M. N. Groot, Oleg Konovalov and Maciej Jankowski

Table S1 Indexed PXRD reflections of $CoReTiO_2$.

Phase	h	k	l	q (Å^{-1})
TiO_2#Anatase	1	0	1	1.79
	1	0	3	2.59
	0	0	4	2.65
	1	1	2	2.70
	2	0	0	3.32
	1	0	5	3.70
	2	1	1	3.77
	2	1	3	4.21
	2	0	4	4.24
	1	1	6	4.61
	2	2	0	4.70
TiO_2#Rutile	1	1	0	1.93
	1	0	1	2.53
	2	0	0	2.74
	1	1	1	2.88

	2	1	0	3.06
	2	1	1	3.72
	2	2	0	3.87
	3	1	0	4.32
	1	1	2	4.66
Co_3O_4	1	1	1	1.34
	2	2	0	2.20
	4	0	0	3.10
	5	1	1	4.03
	4	4	0	4.39
CoO	1	1	1	2.55
	2	0	0	2.95
	2	2	0	4.17
Co-HCP	1	0	0	2.89
	0	0	2	3.07
	1	0	1	3.28
Co-FCC	1	1	1	3.08
	2	0	0	3.52

Table S2 Quantitative phase analysis of $CoReTiO_2$ via Rietveld refinement (Raw Weight Fractions).

Temperature (ºC)	Anatase	Anatase_err	Rutile	Rutile_err	Co_3O_4	Co_3O_4_err	CoO	CoO_err	Co_hcp	hcp_err	Co_fcc	fcc_err
30 ºC	75.1030	0.1300	9.4270	0.0880	15.4700	0.1190	0.0000	0.0000	0.0000	0.0000	0.0000	0.0000
120 ºC	75.5600	0.1560	9.4670	0.0910	13.2530	0.1490	1.7200	0.1810	0.0000	0.0000	0.0000	0.0000
135 ºC	75.6230	0.1530	9.4620	0.0910	12.7180	0.1470	2.1960	0.1720	0.0000	0.0000	0.0000	0.0000
150 ºC	75.6630	0.1510	9.4520	0.0910	12.2100	0.1460	2.6750	0.1660	0.0000	0.0000	0.0000	0.0000
164 ºC	75.7040	0.1480	9.4480	0.0910	11.5610	0.1450	3.2870	0.1580	0.0000	0.0000	0.0000	0.0000
179 ºC	75.7430	0.1460	9.4350	0.0910	10.7680	0.1440	4.0540	0.1520	0.0000	0.0000	0.0000	0.0000
194 ºC	75.7980	0.1440	9.4310	0.0910	9.6690	0.1440	5.1010	0.1460	0.0000	0.0000	0.0000	0.0000
208 ºC	75.9060	0.1440	9.4230	0.0910	8.2810	0.1480	6.3910	0.1390	0.0000	0.0000	0.0000	0.0000
223 ºC	75.9440	0.1490	9.4070	0.0910	6.6580	0.1650	7.9920	0.1320	0.0000	0.0000	0.0000	0.0000
238 ºC	74.6250	0.2070	9.4280	0.0880	6.3370	0.2680	9.6100	0.1290	0.0000	0.0000	0.0000	0.0000
253 ºC	77.6920	0.1280	9.7330	0.0930	0.0000	0.0000	12.5750	0.1150	0.0000	0.0000	0.0000	0.0000
267 ºC	78.2320	0.1320	9.7670	0.0930	0.0000	0.0000	12.0010	0.1190	0.0000	0.0000	0.0000	0.0000
282 ºC	78.2920	0.1420	9.7320	0.0920	0.0000	0.0000	11.3670	0.1320	0.6090	0.1780	0.0000	0.0000
296 ºC	77.9430	0.1880	9.7170	0.0940	0.0000	0.0000	10.2240	0.1410	2.1170	0.1560	0.0000	0.0000
311 ºC	77.7400	0.2180	9.6610	0.0940	0.0000	0.0000	8.8600	0.1560	3.7390	0.2050	0.0000	0.0000
326 ºC	77.1940	0.2390	9.5570	0.0950	0.0000	0.0000	7.2370	0.1630	6.0110	0.2400	0.0000	0.0000
341 ºC	76.3810	0.2560	9.4260	0.0950	0.0000	0.0000	5.3730	0.1630	8.8200	0.2650	0.0000	0.0000

355 ºC	77.5880	0.2370	9.5790	0.0920	0.0000	0.0000	3.7300	0.1410	7.3470	0.3620	1.7550	0.1970
370 ºC	77.3010	0.2480	9.5010	0.0920	0.0000	0.0000	1.7460	0.1200	9.2080	0.4050	2.2430	0.2210
384 ºC	77.0090	0.2720	9.5450	0.0930	0.0000	0.0000	0.0000	0.0000	10.8900	0.4830	2.5560	0.2470
399 ºC	76.8740	0.2990	9.4970	0.0930	0.0000	0.0000	0.0000	0.0000	10.6820	0.5350	2.9470	0.2520
399 ºC	76.4040	0.0670	9.4210	0.0900	0.0000	0.0000	0.0000	0.0000	11.3650	0.1280	2.8110	0.1530
400 ºC	76.5290	0.2840	9.4240	0.0910	0.0000	0.0000	0.0000	0.0000	11.0700	0.5140	2.9770	0.2500

Table S3 Normalized atomic fraction of cobalt derived from Rietveld refinement of $CoReTiO_2$.

Temperature (^{o}C)	Co_3O_4	err	CoO	err	Co-HCP	err	Co-FCC	err
30 ^{o}C	1	0	0	0	0	0	0	0
120 ^{o}C	0.8788	0.0012	0.1212	0.0113	0	0	0	0
135 ^{o}C	0.8435	0.0015	0.1565	0.0104	0	0	0	0
150 ^{o}C	0.8084	0.0019	0.1916	0.0095	0	0	0	0
164 ^{o}C	0.7749	0.0024	0.2251	0.0086	0	0	0	0
179 ^{o}C	0.7091	0.0028	0.291	0.0076	0	0	0	0
194 ^{o}C	0.6373	0.0035	0.3627	0.0066	0	0	0	0
208 ^{o}C	0.5447	0.0046	0.4553	0.0053	0	0	0	0
223 ^{o}C	0.4388	0.006	0.5612	0.0041	0	0	0	0
238 ^{o}C	0.3799	0.0101	0.6201	0.0032	0	0	0	0
253 ^{o}C	0	0	1	0	0	0	0	0
267 ^{o}C	0	0	1	0	0	0	0	0
282 ^{o}C	0	0	0.8675	0.0013	0.1325	0.0167	0	0
296 ^{o}C	0	0	0.7941	0.0023	0.2059	0.0123	0	0
311 ^{o}C	0	0	0.6507	0.004	0.3493	0.0121	0	0
326 ^{o}C	0	0	0.4871	0.0056	0.5129	0.0098	0	0
341 ^{o}C	0	0	0.3239	0.0066	0.6761	0.0066	0	0

355 ºC	0	0	0.1522	0.0081	0.6758	0.0069	0.172	0.0107
370 ºC	0	0	0.1602	0.0065	0.7212	0.0089	0.1727	0.0143
384 ºC	0	0	0.0395	0.0073	0.7734	0.0079	0.1871	0.0144
399 ºC	0	0	0	0	0.8037	0.0066	0.1963	0.0137
399 ºC	0	0	0	0	0.8022	0.0018	0.1978	0.0085
400 ºC	0	0	0	0	0.8046	0.0062	0.1954	0.0135

Table S4 TiO_2-Anatase particle sizes, lattice constants, and phase content during the reduction process corresponding to Figure 5B.

Temperature	TiO_2- Anatase (I 41- a m d)					
	Weight Fraction	Size (nm)	Lattice constants			
			a	c	volume	density
120 ^{o}C	75.560	31.1	3.7801	9.4924	135.636	3.913
179 ^{o}C	75.743	31.1	3.7809	9.4963	135.749	3.909
253 ^{o}C	77.692	31.0	3.7816	9.5026	135.894	3.905
355 ^{o}C	77.588	30.8	3.7828	9.5133	136.129	3.898
399 ^{o}C	76.874	30.9	3.7834	9.5179	136.242	3.895
400 ^{o}C	76.529	30.9	3.7835	9.5182	136.250	3.895

Table S5 TiO_2-Rutile particle sizes, lattice constants, and phase content during the reduction process corresponding to Figure 5B.

Temperature	TiO_2- Rutile (P 42- m n m)					
	Weight Fraction	Size (nm)	Lattice constants			
			a	c	volume	density
120 ºC	9.467	50.9	4.5878	2.956	62.218	4.265
179 ºC	9.435	51.1	4.5892	2.9574	62.287	4.260
253 ºC	9.733	51.1	4.5910	2.9592	63.371	4.254
355 ºC	9.579	51.8	4.5938	2.9614	62.495	4.246
399 ºC	9.497	51.7	4.5953	2.9621	62.550	4.242
400 ºC	9.424	51.8	4.5954	2.9620	62.551	4.242

Table S6 Co_3O_4 particle sizes, lattice constants, and phase content during the reduction process corresponding to Figure 5B.

Temperature	Co_3O_4 (F d - 3 m z)				
	Weight Fraction	Size (nm)	Lattice constants		
			a	volume	density
120 ^oC	13.2530	6.80	8.1074	532.891	6.003
179 ^oC	10.7680	6.9	8.1103	533.473	5.996
253 ^oC	0	/	/	/	/
355 ^oC	0	/	/	/	/
399 ^oC	0	/	/	/	/
400 ^oC	0	/	/	/	/

Table S7 CoO particle sizes, lattice constants, and phase content during the reduction process corresponding to Figure 5B.

Temperature	CoO (F m - 3 m)				
	Weight Fraction	Size (nm)	Lattice constants		
			a	volume	density
120 °C	1.720	3.6	4.2516	76.853	6.476
179 °C	4.054	4.6	4.2595	77.280	6.440
253 °C	12.575	6.1	4.2641	77.531	6.419
355 °C	3.730	6.3	4.2736	78.049	6.377
399 °C	0	/	/	/	/
400 °C	0	/	/	/	/

Table S8 Co-HCP particle sizes, lattice constants, and phase content during the reduction process corresponding to Figure 5B.

Temperature	Co-HCP (P 63- m m c)					
	Weight Fraction	Size (nm)	Lattice constants			
			a	c	volume	density
120 ^{o}C	0	/	/	/	/	/
179 ^{o}C	0	/	/	/	/	/
253 ^{o}C	0	/	/	/	/	/
355 ^{o}C	7.347	2.00	2.5425	4.0797	22.839	8.570
399 ^{o}C	10.682	2.00	2.5455	4.0837	22.915	8.541
400 ^{o}C	11.070	2.00	2.5392	4.0836	22.801	8.584

Table S9 Co-FCC particle sizes, lattice constants, and phase content during the reduction process corresponding to Figure 5B.

Temperature	Co-FCC (F m - 3 m)				
	Weight Fraction	Size (nm)	Lattice constants		
			a	volume	density
120 ^{o}C	0	/	/	/	/
179 ^{o}C	0	/	/	/	/
253 ^{o}C	0	/	/	/	/
355 ^{o}C	1.755	6.4	3.5676	45.409	8.620
399 ^{o}C	2.947	5.2	3.5671	45.388	8.640
400 ^{o}C	2.977	5.3	3.5644	45.285	8.644

Table S10 Quantitative speciation from XANES via linear combination fitting (LCF).

Temperature (^oC)	Co_3O_4 frac	error	CoO frac	error	Co fraction	error	chi2	chi2-red
30 ^oC	1.0000	0.0376	0.0000	0.0224	0.0000	0.0395	0.0000	0.0000
128 ^oC	0.2270	0.0435	0.7773	0.0486	0.0091	0.0364	100.1055	1.2834
143 ^oC	0.2852	0.0402	0.7148	0.0446	0.0000	0.0272	92.3671	1.1842
157 ^oC	0.1956	0.0391	0.8044	0.0421	0.0000	0.0200	116.3688	1.4919
172 ^oC	0.1537	0.0404	0.8463	0.0432	0.0000	0.0387	63.4446	0.8134
187 ^oC	0.2271	0.1843	0.7729	0.1639	0.0000	0.2267	83.7944	1.0743
201 ^oC	0.1770	0.0408	0.8221	0.0436	0.0000	0.0177	139.8243	1.7926
216 ^oC	0.1068	0.1582	0.8932	0.0874	0.0000	0.1794	108.3619	1.3893
231 ^oC	0.0912	0.2045	0.9088	0.3347	0.0000	0.4077	86.6671	1.1111
245 ^oC	0.0000	0.0348	1.0000	0.0428	0.0000	0.0363	0.0000	0.0000
260 ^oC	0.0812	0.1545	0.8695	0.0724	0.0494	0.1686	122.7073	1.5732
275 ^oC	0.1030	0.2303	0.8403	0.1966	0.0566	0.3579	65.8399	0.8441
289 ^oC	0.0576	0.1916	0.7480	0.1734	0.1944	0.2991	104.4773	1.3395
304 ^oC	0.0851	0.0388	0.6750	0.0413	0.2399	0.0401	87.3870	1.1203
319 ^oC	0.0788	0.0842	0.5631	0.0532	0.3581	0.0908	92.4970	0.1859

334 °C	0.1073	0.0179	0.4307	0.0047	0.4620	0.0217	99.3531	1.2738
348 °C	0.1084	0.0058	0.3916	0.0026	0.5000	0.0116	106.1767	1.3612
363 °C	0.0661	0.0036	0.2426	0.0017	0.6913	0.0102	104.4959	1.3397
377 °C	0.1024	0.0036	0.0792	0.0009	0.8184	0.0100	76.0643	0.9752
392 °C	0.0627	0.0096	0.0000	0.0021	0.9373	0.0143	88.0158	1.1284
400 °C	0.0621	0.0289	0.0000	0.0055	0.9379	0.0345	67.4242	0.8644
400 °C	0.0000	0.0011	0.0000	0.0005	1.0000	0.0088	0.0000	0.0000
400 °C	0.0171	0.0098	0.0416	0.0021	0.9431	0.0145	92.3934	1.1845

scan_0010_fit.png

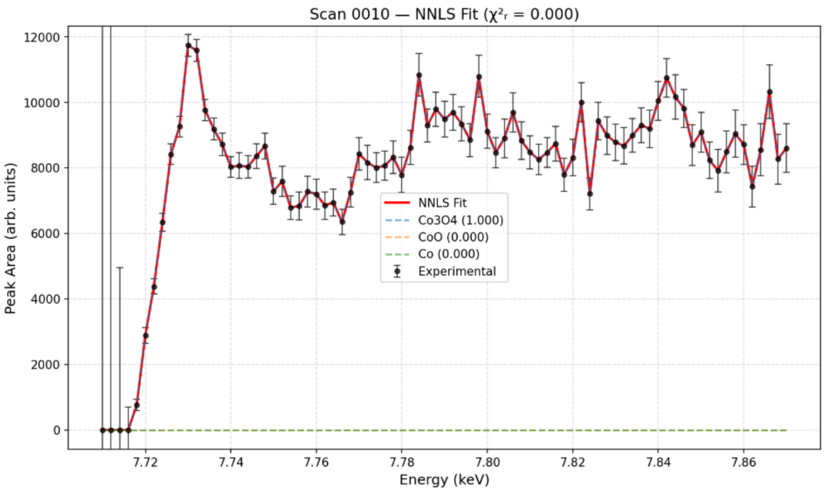

scan_0054_fit.png

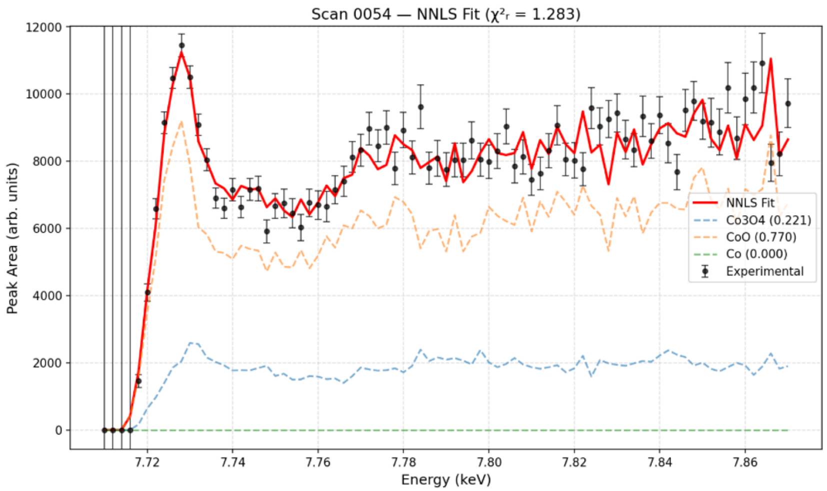

scan_0058_fit.png

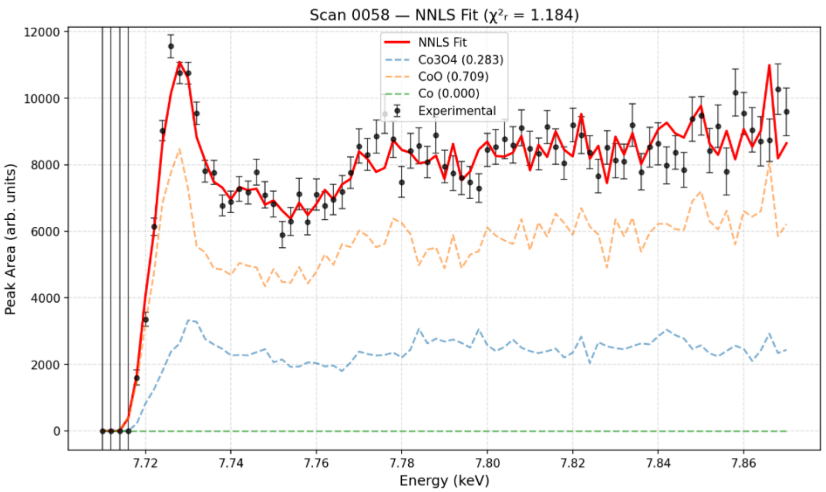

scan_0062_fit.png

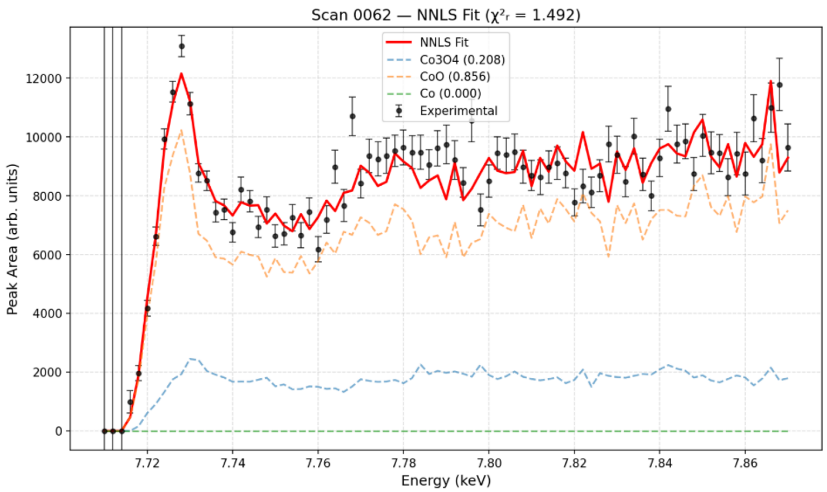

scan_0066_fit.png

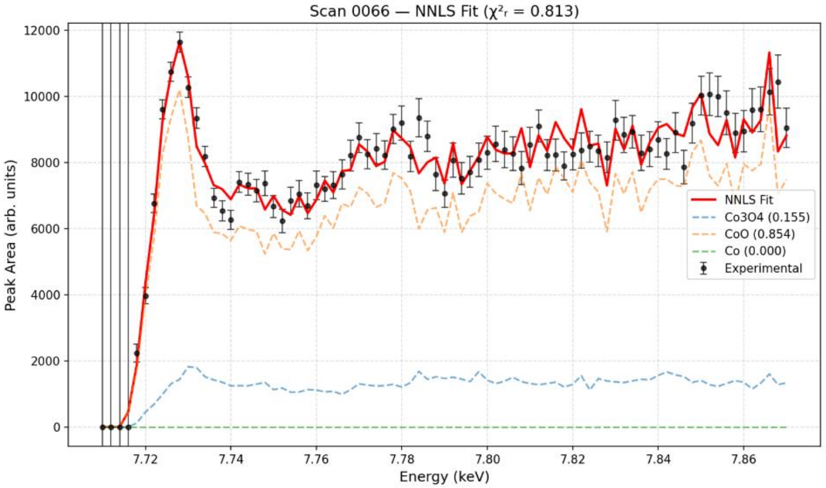

scan_0070_fit.png

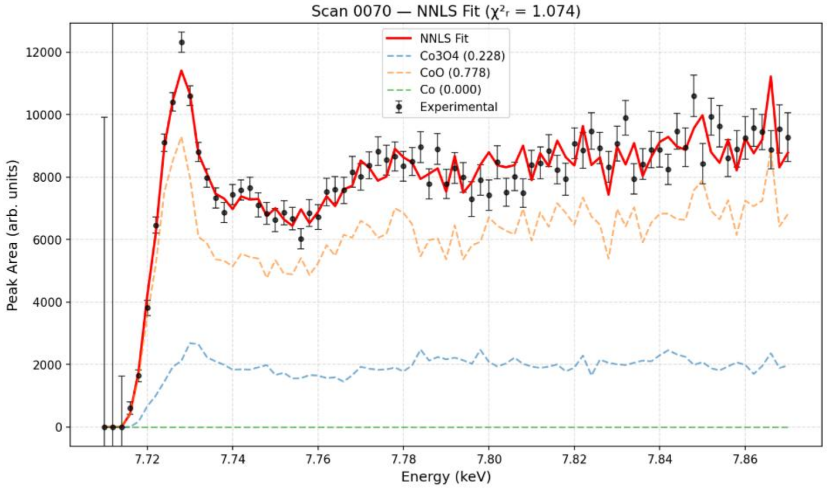

scan_0074_fit.png

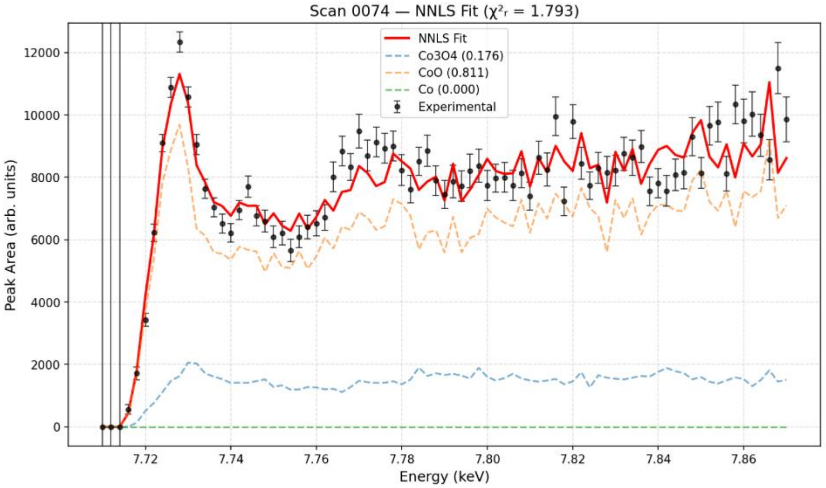

scan_0078_fit.png

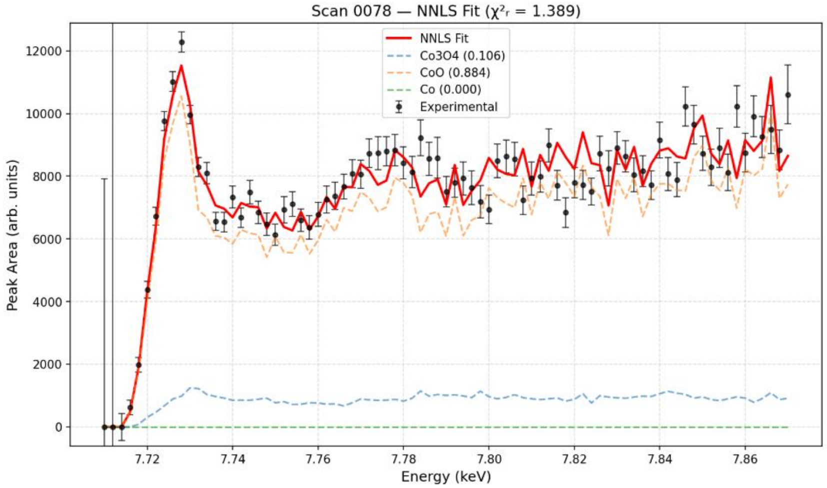

scan_0082_fit.png

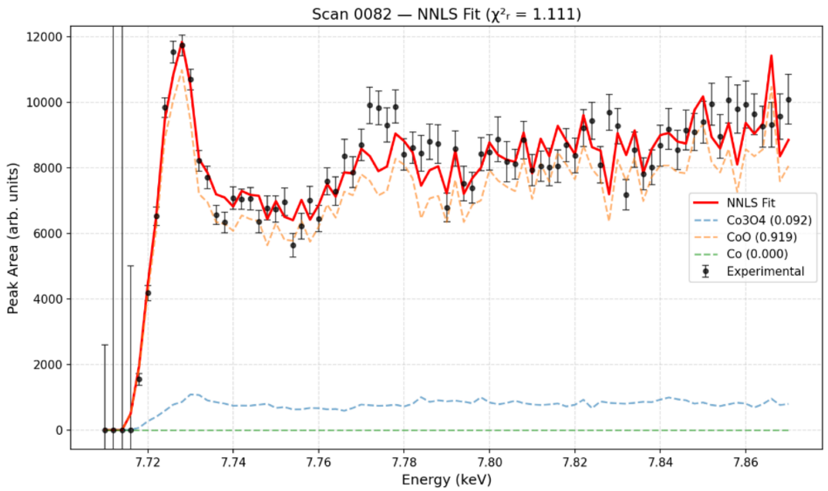

scan_0086_fit.png

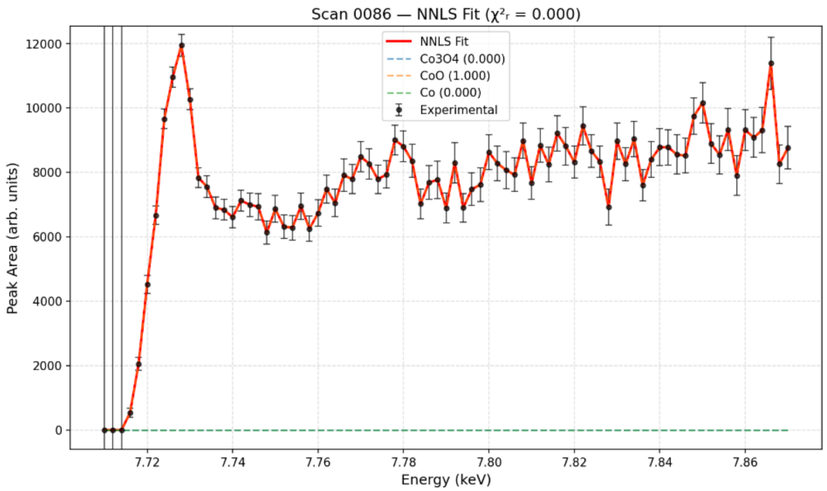

scan_0090_fit.png

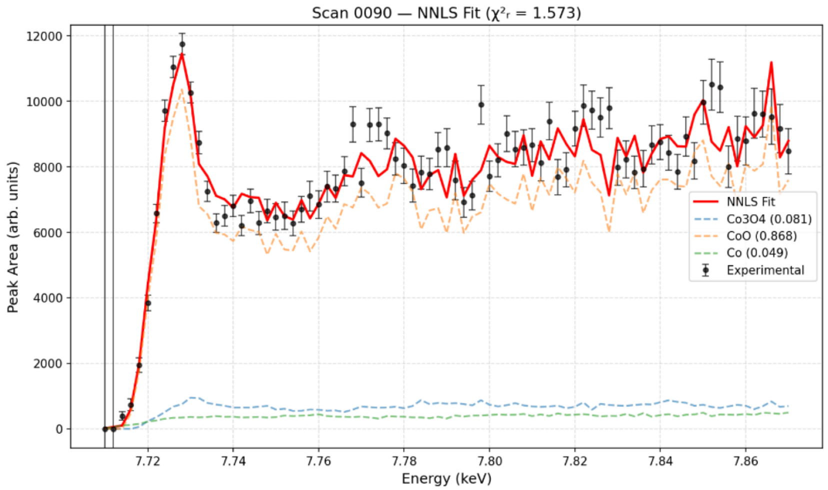

scan_0094_fit.png

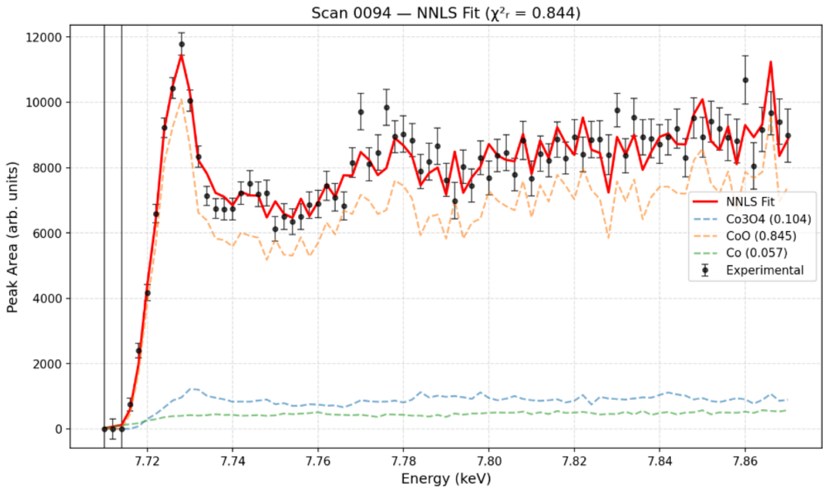

scan_0098_fit.png

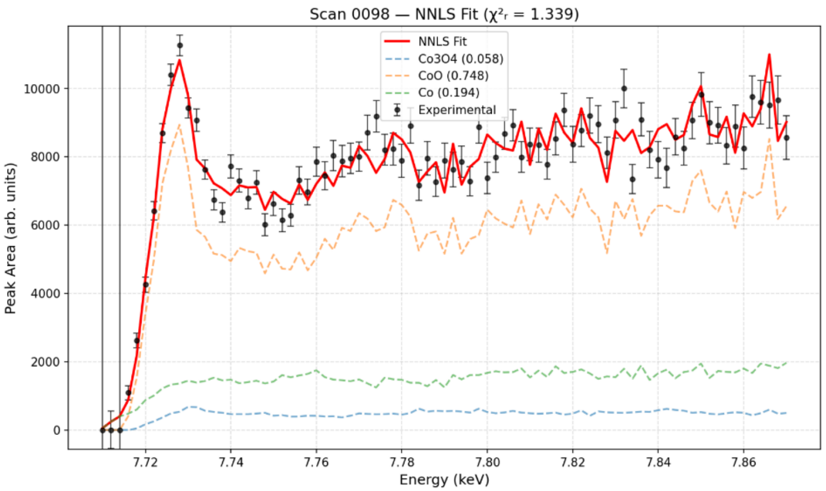

scan_0102_fit.png

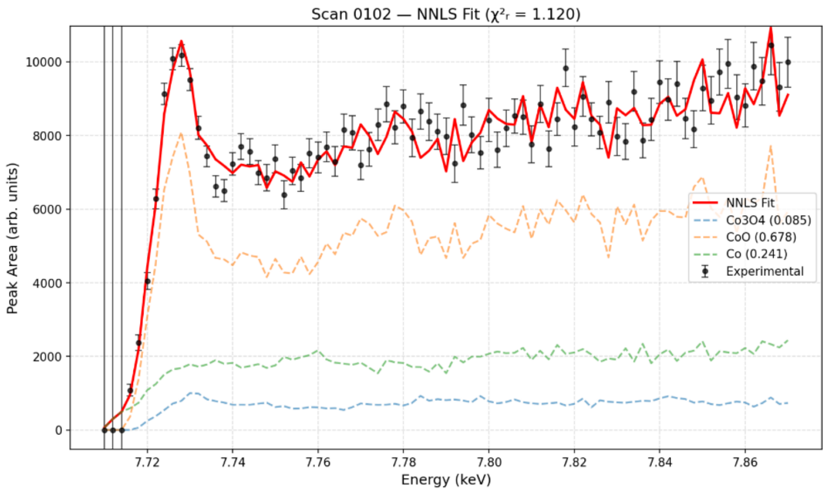

scan_0106_fit.png

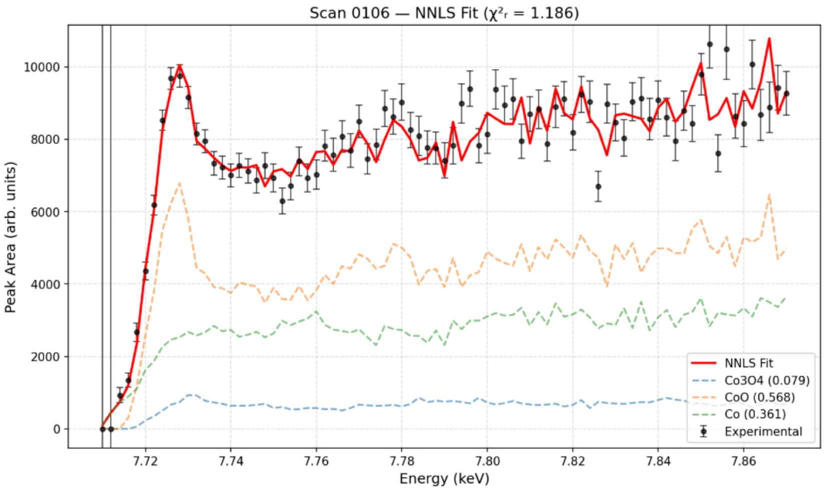

scan_0110_fit.png

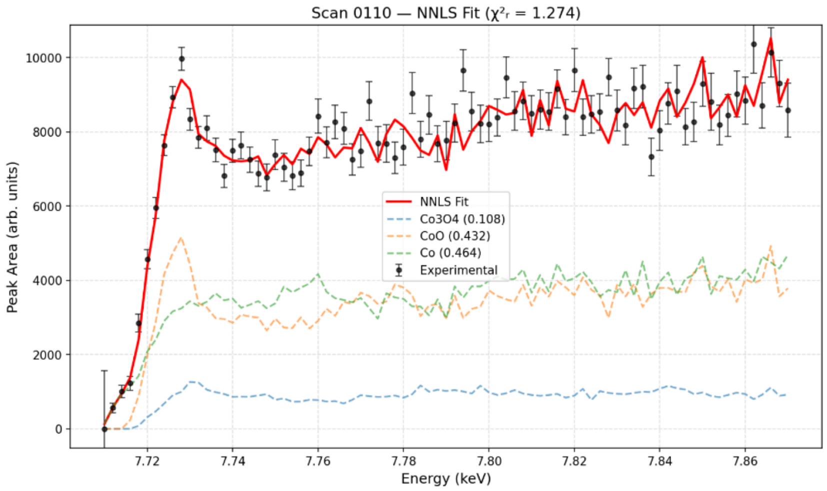

scan_0114_fit.png

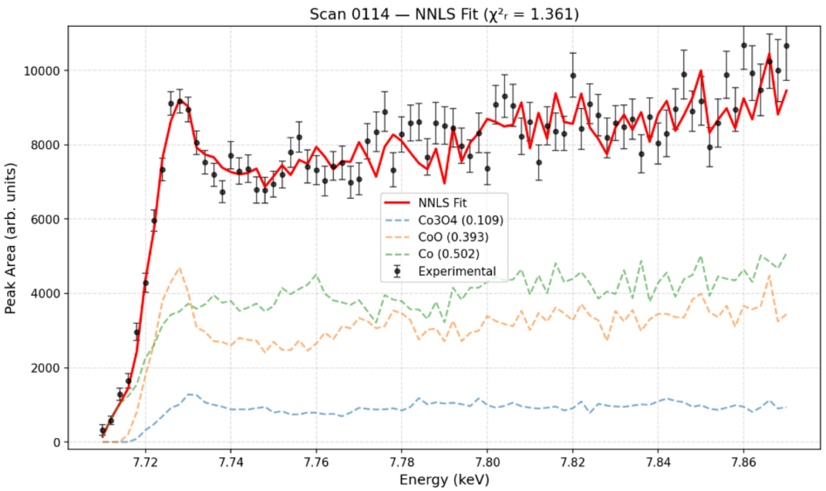

scan_0118_fit.png

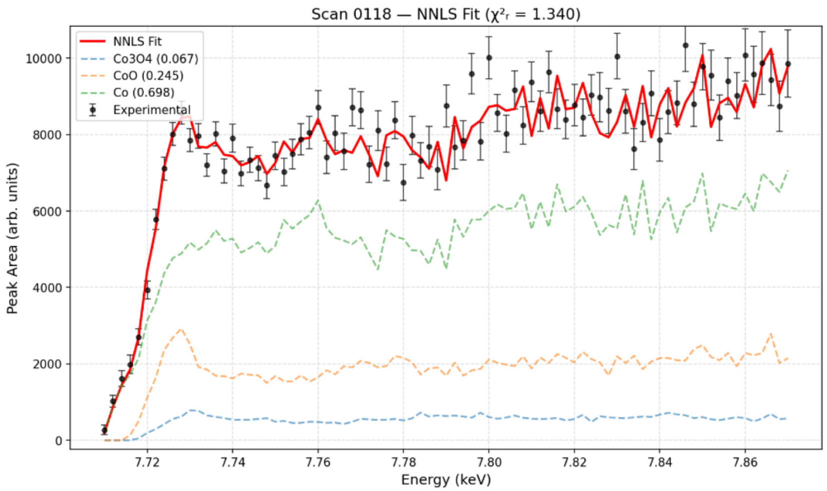

scan_0122_fit.png

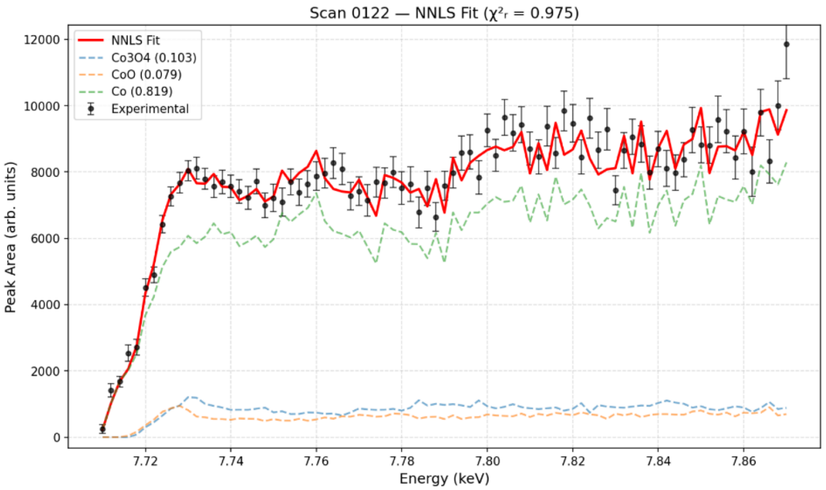

scan_0126_fit.png

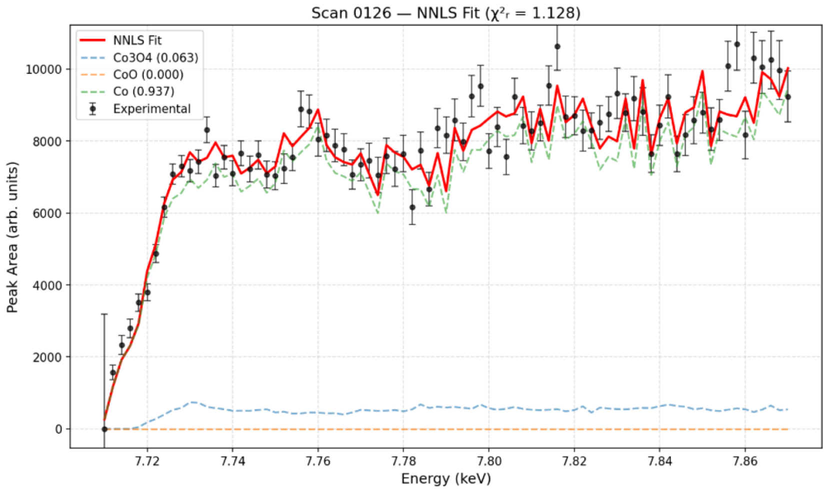

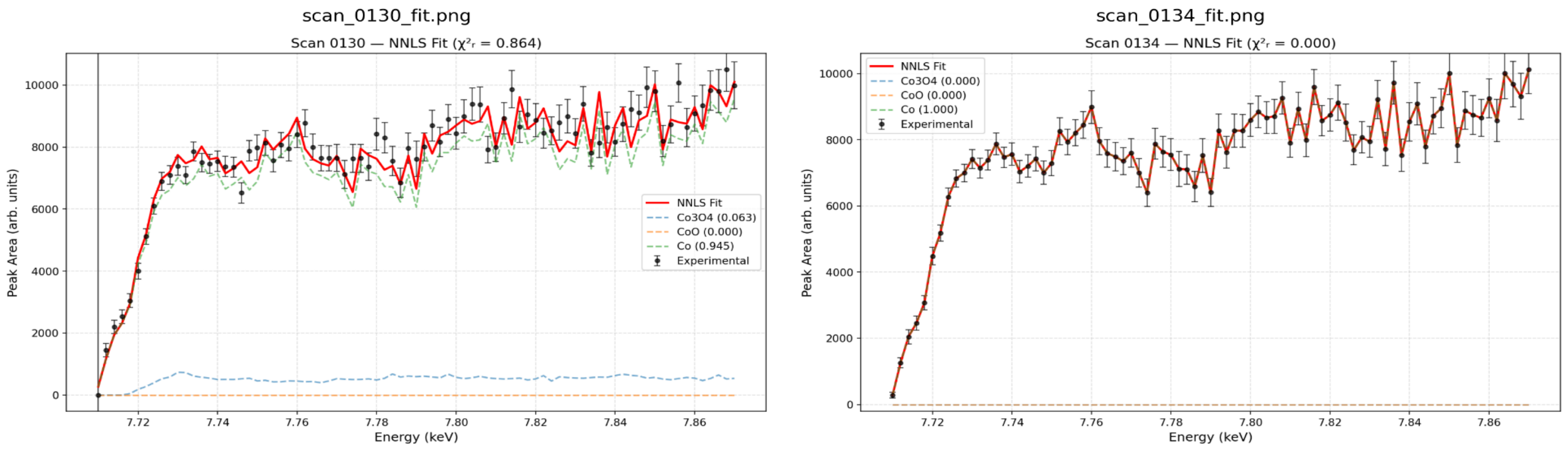

Figure S1 LCF fitting results for each scan using the NNSL method. Experimental spectra (dots) are fitted with a linear combination of reference spectra. The χ^2 values indicate the goodness of fit.

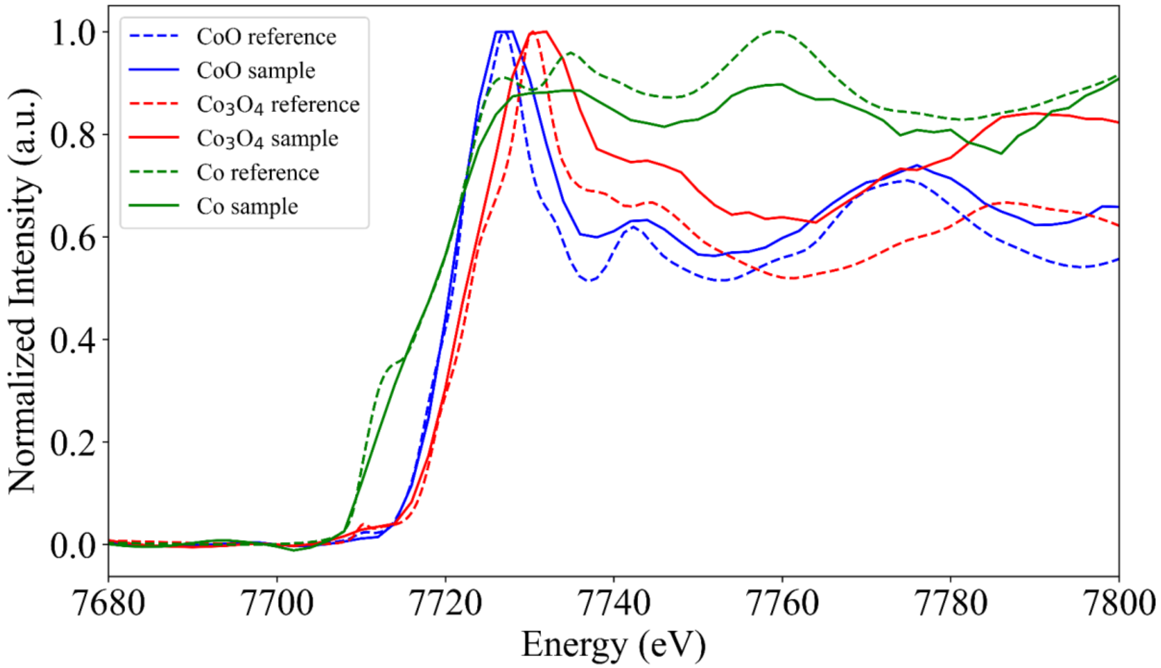

Figure S2 The comparison of experimental reference samples with the standard sample measured at the Beamline for Materials Measurement (BMM), Brookhaven National Laboratory using an ionization chamber detector in transmission mode.

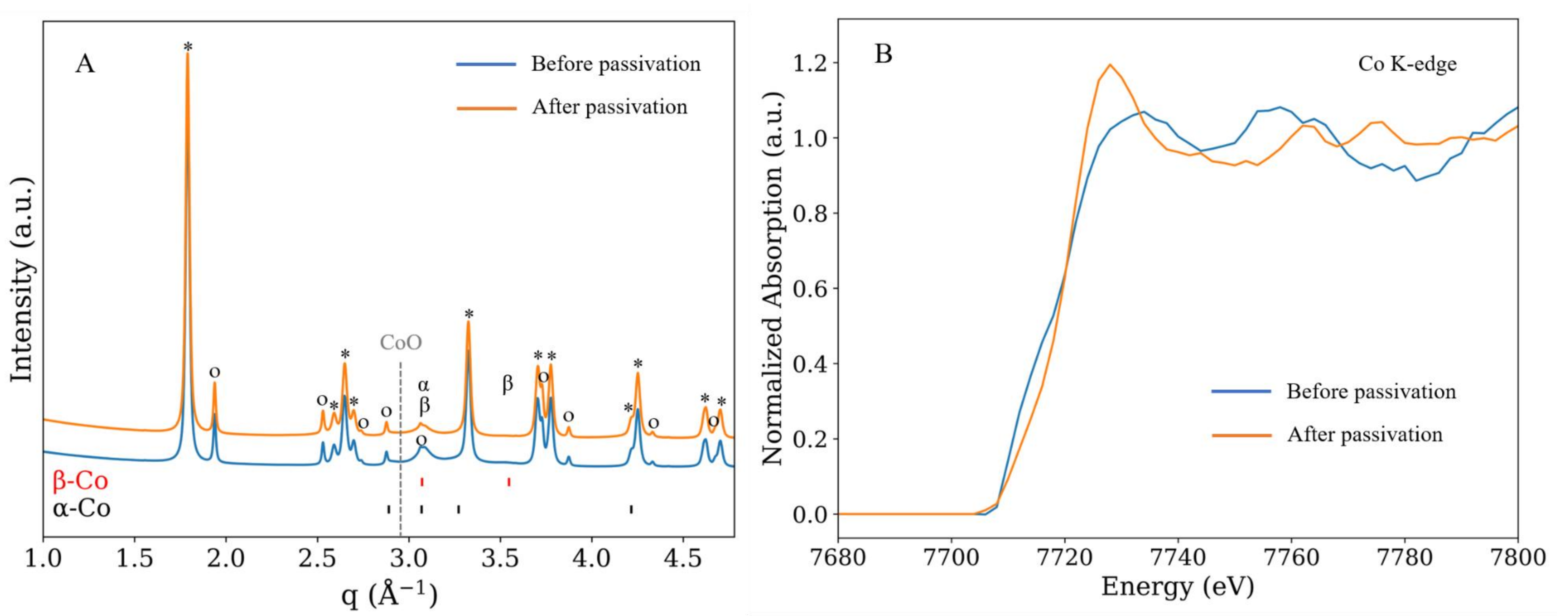

Figure S3 (A) PXRD patterns of the sample before and after passivation, showing no detectable CoO-related peaks. (B) XANES spectra before and after passivation, revealing the formation of CoO after passivation compared to before passivation.
